# High‐Performance Sunlight‐Induced Polymerized Hydrogels and Applications in 3D and 4D Printing

**DOI:** 10.1002/smll.202411888

**Published:** 2024-12-18

**Authors:** Ji Feng, Zheng Liu, Tong Gao, Didier Gigmes, Fabrice Morlet‐Savary, Michael Schmitt, Celine Dietlin, Tatiana Petithory, Laurent Pieuchot, Jing Zhang, Wenpeng Shan, Pu Xiao, Frédéric Dumur, Jacques Lalevée

**Affiliations:** ^1^ Université de Haute‐Alsace CNRS IS2M UMR7361 Mulhouse F‐68100 France; ^2^ Université de Strasbourg Strasbourg 67000 France; ^3^ Aix Marseille Univ CNRS ICR UMR 7273 Marseille F‐13397 France; ^4^ Future Industries Institute University of South Australia Mawson Lakes SA 5095 Australia; ^5^ State Key Laboratory of High‐Performance Ceramics and Superfine Microstructure Shanghai Institute of Ceramics Chinese Academy of Sciences Shanghai 200050 P. R. China

**Keywords:** 3D printing, 4D printing, sunlight‐induced photopolymerization, sunlight polymerized hydrogels, water‐soluble photoinitiator

## Abstract

Currently, there are only few reports on water‐soluble photoinitiating systems. In this study, a highly water‐soluble organic dye i.e. sodium (E)‐3,3′‐((4‐(2‐(3‐methylbenzo[*d*]thiazol‐3‐ium‐2‐yl)vinyl)phenyl)azanediyl)dipropionate iodide, was synthesized and served as a photoinitiator. Notably, this water‐soluble initiator, at a low concentration of just 0.01 wt%, demonstrates a high photoinitiation ability, with some hydrogel formulations achieving nearly 100% double bond conversion under sunlight. Photopolymerization kinetics were monitored using Real‐Time Fourier Transform Infrared. To explore the complex chemical principles of radical polymerization, UV‐visible absorption and fluorescence spectroscopy, steady‐state photolysis, fluorescence quenching experiments and cyclic voltammetry were employed to gain a comprehensive understanding of the photochemical mechanism involved. Additionally, several characteristics of the synthesized hydrogels were also investigated i.e. the water content, the water swelling, and the volume swelling. In addition to their excellent photoinitiation capabilities, the hydrogel formulations developed in this study also supported 3D printing. 3D objects with smooth surface and a high spatial resolution could be successfully printed using direct laser writing. The fabricated hydrogels could reversibly change of shape in response to water (adding or removing water), enabling successful 4D printing behavior. Furthermore, the efficient photoinitiation ability of the water‐soluble formulations opens new avenues for sunlight‐polymerized hydrogels and potential applications in bioprinting.

## Graphical abstract

In this study, a highly water‐soluble organic dye is synthesized and served in high performance photoinitiating systems. In addition to their excellent photoinitiation capabilities, the hydrogel formulations developed in this study also support 3D printing. The fabricated hydrogels could reversibly change of shape in response to water, enabling successful 4D printing behavior.

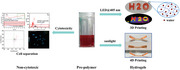

## 1 Introduction

Hydrogels are a type of crosslinked 3D polymer networks characterized by their high water content, allowing them to retain large amounts of water without dissolving.^[^ ^1^, ^2^, ^3^, ^4^ ^]^ Hydrogels can swell in water many times their weight in the dry state.^[^ ^5^, ^6^ ^]^ Hydrogels usually^[^ ^7^ ^]^ exhibit a high permeability and an excellent biocompatibility due to a high water content and softness, enabling them to simulate natural human tissues. This makes them highly attractive in the field of biomedical materials.^[^ ^5^, ^8^, ^9^, ^10^ ^]^ However, most hydrogels lack shape memory properties and cannot change shape in response to environmental triggers (e.g., water and temperature).^[^ ^7^ ^]^ Therefore, it is particularly important to develop intelligent hydrogels with shape memory functions.

Compared with traditional methods such as solvent and thermal polymerization, visible‐light‐induced polymerization is an ideal method for preparing hydrogels.^[^ ^11^, ^12^, ^13^ ^]^ Photopolymerization offers the advantages of simple operation, low cost, rapid curing at room temperature or physiological temperature, and low heat generation. Importantly, it is an environmentally friendly technology.^[^ ^14^, ^15^ ^]^ Photopolymerization also allows for curing exclusively in the irradiation area of the light source, providing an excellent temporal and spatial control over the produced objects.^[^ ^16^, ^17^, ^18^ ^]^ For the photopolymerization systems, the light source is a crucial factor. Compared with the traditional high‐energy light sources (e.g., UV light or γ rays), long‐wavelength visible light (>400 nm) is generally recognized as a safer initiation light source, as it poses less potential harm to active substances such as cells and the environment.^[^ ^19^, ^20^ ^]^ However, the most ideal light source remains sunlight. As a renewable energy source, sunlight aligns well with the principle of sustainable chemistry. Additionally, its low light intensity is less harmful,^[^ ^14^, ^21^, ^22^ ^]^ laying the foundation for the application of sunlight‐polymerized hydrogels in the biomedical field. Nevertheless, there are currently few reports on sunlight‐induced hydrogels, highlighting the importance of developing efficient methods for hydrogel polymerization using sunlight.

Another important factor in photopolymerization is the photoinitiator. In recent years, photoinitiating systems in water have attracted a great attention^[^ ^23^, ^24^, ^25^, ^26^ ^]^ because the obtained polymers can offer better biocompatibility. Additionally, with increasing expenditure on environmental protection in various countries, photopolymerization in water has been identified as an environmentally friendly method. Its application scope can be extended to fields such as food packaging materials and biomedicine.^[^ ^27^ ^]^ Currently, there are only a few water‐soluble photoinitiators and they are often cytotoxic, which especially hinders their applications in tissue engineering through photopolymerization.^[^ ^24^, ^28^, ^29^ ^]^ Therefore, designing water‐soluble photoinitiators with excellent performance and low toxicity is a crucial focus.

**Scheme 1:**
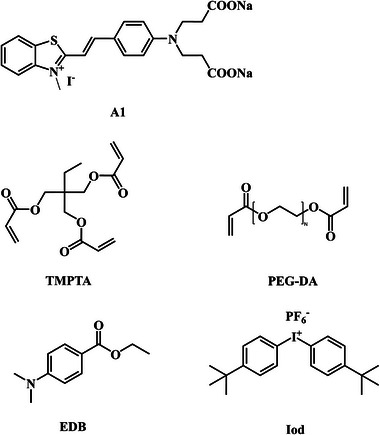
Chemical structures of dye‐A1, additives, and monomers.

Herein, we designed and synthesized a water‐soluble photoinitiator, as shown in **Scheme** **1**. Efficient photoinitiating systems could be developed with this structure which were able to induce the synthesis of polyethylene glycol diacrylates (average *M*n = 575 g mol^−1^), PEGDA‐based hydrogels under visible light LED@405 nm or even sunlight. It demonstrated that, in the presence of a low content of photoinitiator (only 0.01 wt%, much lower than those used in the previous studies,^[^ ^30^, ^31^ ^]^ the polymerization of hydrogels could be efficiently initiated and polymerized at 405 nm or under sunlight. Then, the relevant photochemical mechanism was studied in detail using a range of analytical techniques, including UV–visible light spectroscopy, fluorescence spectroscopy, steady‐state photolysis, and fluorescence quenching. Additionally, the properties of the synthesized hydrogels, such as the water content and the swelling behavior, were investigated. Furthermore, 3D printing experiments of the hydrogels were successfully conducted, achieving high‐resolution 3D objects. Notably, these 3D objects exhibited reversible shape memory functions, which could be triggered by water, thereby enabling the application of 4D printing.

## 2 Results and Discussion

### 2.1 Synthesis of Dye‐A1

**Scheme 2:**
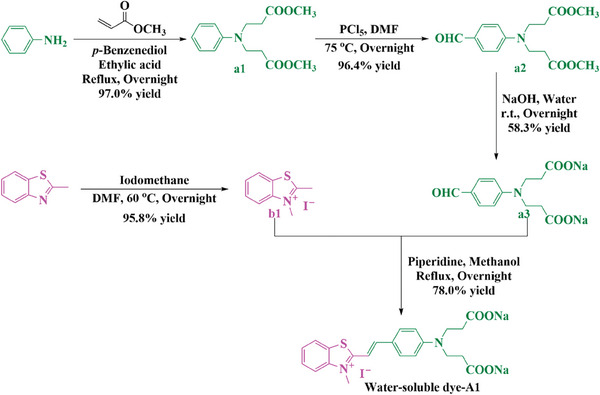
Synthetic routes to dye‐A1.

The dye‐A1 investigated in this work were synthesized through a multistep synthesis approach (see **Scheme** **2**). Notably, the synthesis of water‐soluble dye‐A1 started with aniline and 2‐methylbenzo[*d*]thiazole. First, aniline, methyl acrylate, acetic acid, and hydroquinone were refluxed overnight to obtain a1 with a reaction yield of 97%. Phosphorus pentachloride was added slowly to DMF (100 mL). After stirring at 0 °C and room temperature for 1 h respectively, 3,3′‐(phenylazanediyl)dipropionate (a1) was slowly added. After stirring at 75 °C overnight and then cooled to room temperature, the solution was poured in ice water and neutralized with 2.5% m KOH. The solution was subsequently washed with DCM and dried over MgSO_4_. Compound a2 could be isolated in 61% yield. Synthetic procedures and characterizations were consistent with those previously reported in the literature.^[^ ^32^ ^]^ 3,3′‐((4‐Formylphenyl)azanediyl)dipropionate (a2) and NaOH were added to water and the resulting solution was stirred at room temperature overnight. After most of the water was removed by rotary evaporation, the red solid compound (a3, 58.3% yield) was recrystallized from ethanol. Parallel to this, 2‐methylbenzo[*d*]thiazole and iodomethane were added to DMF (10 mL) and the solution was stirred at 60 °C overnight. After cooling to room temperature, ethyl acetate was added to the solution and the solution was filtered to collect the precipitate. After recrystallizing three times using ethyl acetate, the pure product was obtained and b1 could be isolated in 95.8% yield. Finally, piperidine and b1 were added into a mixture of 2,3‐dimethylbenzo[*d*]thiazol‐3‐ium iodide in methanol (20 mL) and 3,3′‐((4‐formylphenyl)azanediyl)dipropionate (a3) at room temperature, and the solution was refluxed overnight. After cooling to room temperature, a pure red‐brown compound was obtained (dye‐A1, 78.0% yield) by filtering and washing with cold methanol and then with diethyl ether.

### 2.2 Study on Cytotoxicity of Dye‐A1

**Figure 1:**
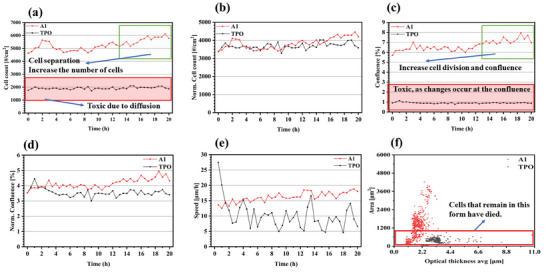
a) Average “cell count” for different treatments. b) Average “Norm. cell count” for different treatments. c) Average “Confluence” for different treatments. d) Average “Norm. Confluence” for different treatments. e) Average “Speed” for different treatments. f) Cells morphology of TPO and dye‐A1.

The toxicity of dye‐A1 was evaluated by cell viability, cytotoxicity, apoptosis and cell morphology through DMEM culture medium and continuous monitoring for 20 h, and compared with the potential toxicity of commercial initiator TPO. Firstly, we continuously measured the average “cell count” of different treatments for 20 h (once every half hour) (see **Figure** **1**a,b). As shown in Figure 1a, the red line represents the cell counts of dye‐A1 within 20 h, and the black line represents TPO. It was evident that the cell division occurred in the culture medium containing dye‐A1, leading to a significant increase in cell numbers. In contrast, TPO was toxic due to diffusion, resulting in cell depth, which caused the cell count to remain almost unchanged within 20 h. Figure 1c,d,f also shows similar results. Dye‐A1 increased cell division and fusion because of its excellent cell compatibility, while TPO caused cell apoptosis because of its toxicity (see Figure 1f). Then, we measured the cell viability through the movement speed and track of cells, as shown in Figure 1e, which was the average “speed” of different treatments. The speed of cells in the medium containing TPO decreased obviously within 2 h, while the movement speed of cells in the medium containing dye‐A1 increased slightly. We can see the difference of cell vitality more intuitively through the track of a single cell, as shown in Figure S1 (Supporting Information), which shows the track of a single cell in the culture medium containing TPO. It can be seen that the cells have already died, with only few cells remaining visible under the Holomonitor (see Figure S2, Supporting Information). As shown in Figure S3 (Supporting Information), the track of single cell movement in the culture medium containing dye‐A1 shows that the cells had vitality, and the number of cells was significantly higher than that of the control TPO (see Figure S4, Supporting Information). The above results show that dye‐A1 did not cause any cytotoxicity. Therefore, dye‐A1 can support the growth, proliferation, and survival of cells, demonstrating excellent biocompatibility.

### 2.3 Photopolymerization Kinetics and Chemical Mechanisms of Dye‐A1

#### 2.3.1 Light Absorption Properties of Dye‐A1 and Steady‐State Photolysis of Dye‐A1‐Alone, Dye‐A1/Iod, Dye‐A1/EDB, and Dye‐A1/Iod/EDB Photoinitiating Systems

**Figure 2:**
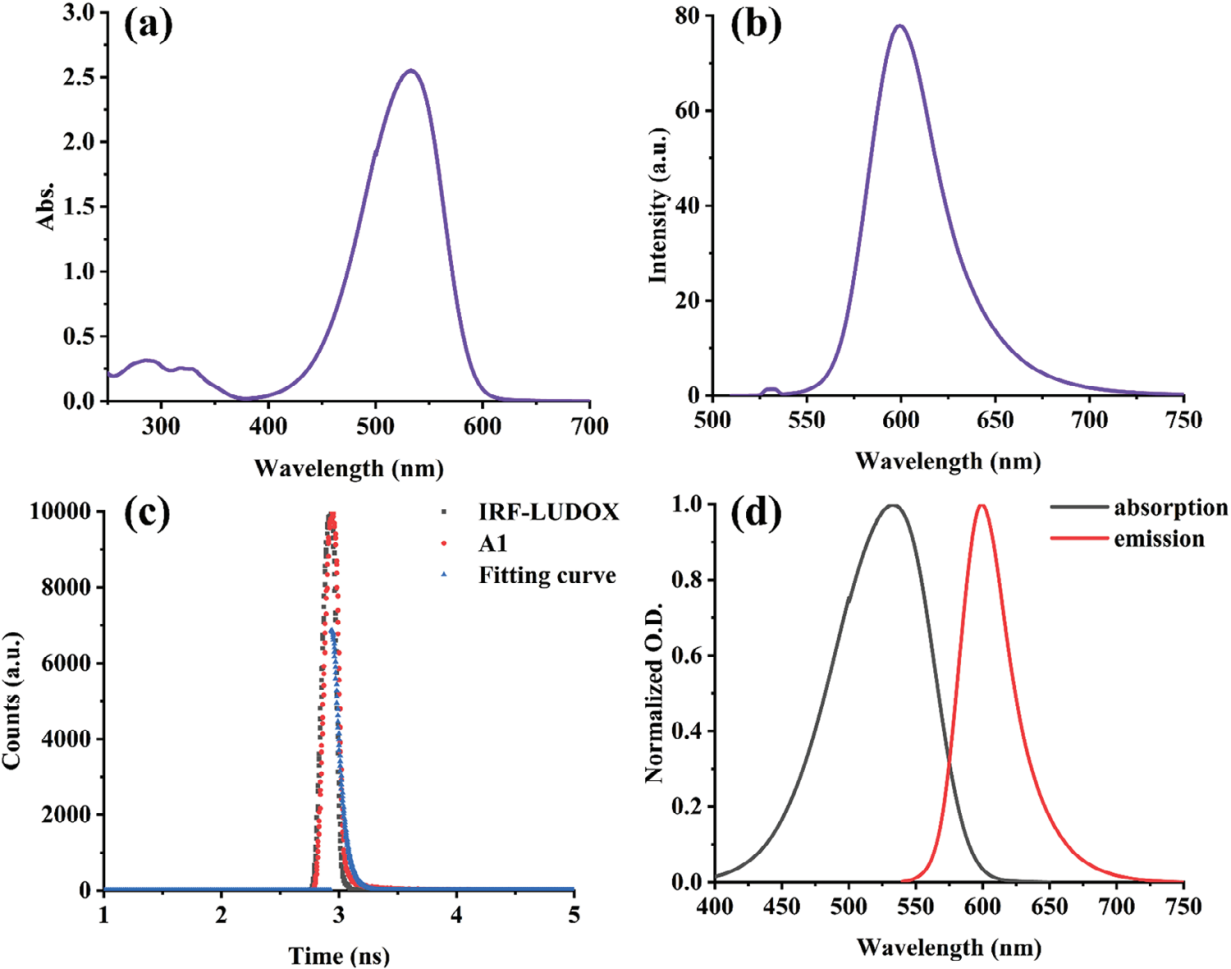
a) UV–visible absorption spectrum, b) fluorescence spectrum, c) fluorescence lifetime, and d) singlet state energy determination of A1 in methanol.

The light absorption of dye‐A1 in methanol is shown in **Figure** **2**a. Obviously, dye‐A1 exhibits a broad absorption extending over the UV–visible region, and the maximum absorption wavelength (*λ*_max_) is located at 530 nm, while the molar extinction coefficient of *λ*_max_ is 51 000 M^−1^cm^−1^. The absorption wavelength, the maximum molar extinction coefficient (*ɛ*_max_) and the molar extinction coefficient at 405 nm (*ɛ*_405_ _nm_) of dye‐A1 are summarized in **Table** **1**. Since the solar energy has a strong radiation between 400 and 600 nm,^[^ ^33^ ^]^ and the *λ*_max_ of dye‐A1 falls within this range, dye‐A1 was chosen as a suitable photosensitizer for sunlight‐induced polymerization. The results in Section 2.3.4 also confirmed that dye‐A1 demonstrated a high photoinitiation ability as a photoinitiator in free radical polymerization. In particular, it can be used for the synthesis of hydrogels under sunlight. Such a performance can hardly be obtained with a commercial photoinitiator.

**Table 1:** Light absorption characteristics of A1.

*λ*_max_ [nm]	*ɛ*_max_ [M^−1^cm^−1^]	*ɛ*_405_ [M^−1^cm^−1^]	Fluorescence lifetime [ns]
530	51 000	1100	0.58
330	4800		
290	6200		

**Figure 3:**
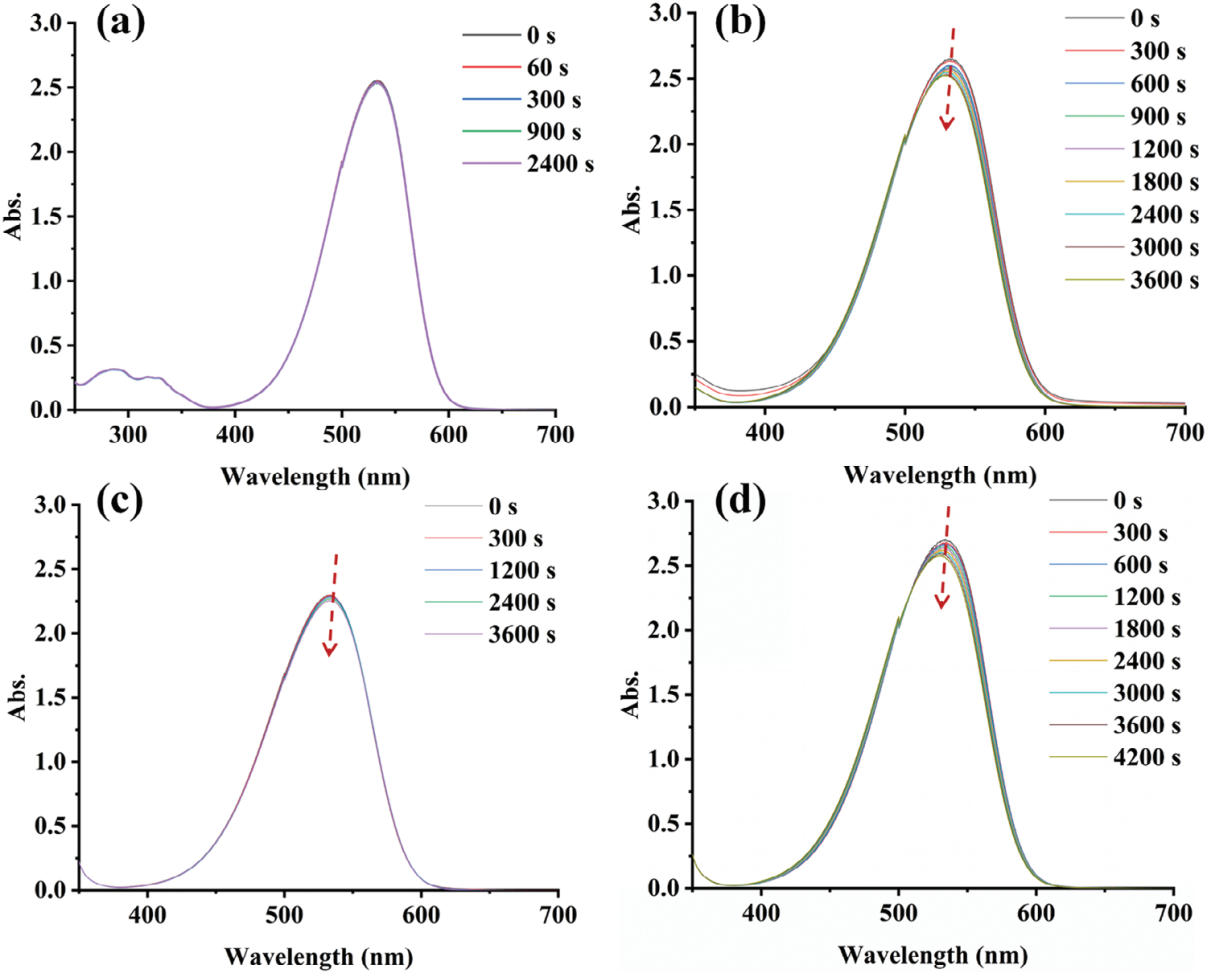
Steady state photolysis of a) A1‐alone. b) A1/Iod. c) A1/EDB. d) A1/Iod/EDB.

To reveal the chemical mechanism in the photopolymerization process comprehensively, the steady state photolysis reaction between dye‐A1 and additives (EDB and Iod) were investigated in methanol under LED@405 nm. As shown in **Figure** **3**, the photolysis of dye‐A1 alone (Figure 3a), dye‐A1/Iod (Figure 3b), dye‐A1/EDB (Figure 3c), and dye‐A1/EDB/Iod (Figure 3d) over a specific period of time is presented. At the same time, the changes during photolysis are shown in Figure S5 (Supporting Information). For the single dye‐A1, no photolysis was observed, demonstrating that it has a good photostability. In the case of dye‐A1/Iod, dye‐A1/EDB, and dye‐A1/EDB/Iod, a clear decrease in absorption peak intensity was observed within 1 h. It indicates that dye‐A1 can react with additives, and the specific reaction process will be discussed in the following sections.

#### 2.3.2 Fluorescence Properties (i.e., Fluorescence Spectra, Fluorescence Excited State Lifetimes, Fluorescence Quenching Experiments) and Electron Transfer Reaction for Dye‐A1

Another important factor for studying the chemical mechanism is the fluorescence properties of dye‐A1 and the efficiency of the electron transfer reactions with additives. The fluorescence spectrum of dye‐A1 is shown in Figure 2b. It can be seen that dye‐A1 showed a strong fluorescence in the range of 550–700 nm, but its fluorescence excited state lifetime was only 0.58 ns (see Figure 2c and Table 1). In particular, combining the UV–visible absorption and fluorescence spectra of dye‐A1 (see Figure 2d), the singlet energy (*E*_S1_) can be calculated from the intersection of their normalized spectra. And the Δ*G*_S1_ of electron transfer between dye‐A1 and additives is listed in **Table** **2**. In addition, the oxidation‐reduction potential of dye‐A1 was measured by cyclic voltammetry as shown in Figure S6 (Supporting Information). The oxidation potential was recorded as *E*_ox_, and the reduction potential was recorded as *E*_red_. The values of *E*_ox_ and *E*_red_ are summarized in Table 2.

**Table 2:** Parameters of the chemical mechanisms associated with dye‐A1 in methanol.

	Dye‐A1
LUMO	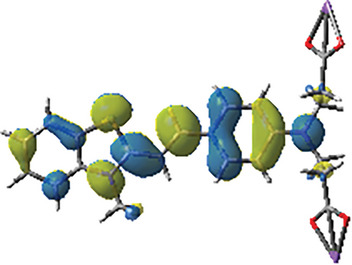
HOMO	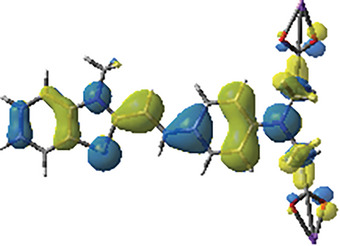
*E*_ox_ (V)	0.42
*E*_red_ (V)	−0.61
*E*_S1_ (eV)	2.17
Δ*G* ^S1^_EDB_ (eV)	−0.56
Δ*G* ^S1^_Iod_ (eV)	−1.05
*E*_T1_ (eV)	1.46
Δ*G* ^T1^_EDB_ (eV)	0.15
Δ*G* ^T1^_Iod_ (eV)	−0.34

**Figure 4:**
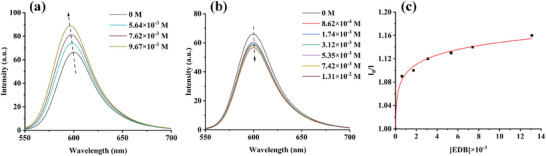
Fluorescence quenching of a) dye‐A1/Iod and b) dye‐A1/EDB in methanol. Stern–Volmer treatment for fluorescence quenching of c) dye‐A1/EDB.

In order to figure out the reaction between dye‐A1 and additives intuitively, the fluorescence quenching experiments of dye‐A1/EDB and dye‐A1/Iod in methanol were carried out. The interaction between dye‐A1/Iod and dye‐A1/EDB resulted in the change of fluorescence emission intensity, which are shown in **Figure** **4**a,b respectively. From Figure 4a, the fluorescence intensity of dye‐A1 and Iod system gradually increased with higher concentrations of Iod, which indicated the system can produce new fluorescent substances during light irradiation.^[^ ^34^ ^]^ From Figure 4b, a quenching reaction can occur between dye‐A1 and EDB, so that the fluorescence intensity gradually decreased. In particular, as shown in Figure 4c, the nonlinear relationship curve of fluorescence spectrum fitting more clearly proved that intramolecular quenching process, collision quenching and static quenching may coexist.^[^ ^35^ ^]^ This was helpful to understand the chemical mechanism in photopolymerization. The above findings indicated that there was an interaction between dye ‐A1 and additives.

#### 2.3.3 Summary of the Photoinitiation Mechanisms and the Theoretical Calculations of Dye‐A1

To further evaluate the photopolymerization mechanism, the triplet excited state energy (*E*_T1_) of dye‐A1 as a crucial theoretical parameter was investigated. The frontier molecular orbitals and *E*_T1_ of dye‐A1 were calculated at B3LYP/6‐31G* level. Herein, the theoretical calculation of *E*_T1_ was carried out by Gaussian 03, and the geometric structure of the ground state was optimized, and the energy levels and electron distribution of HOMO and LUMO were estimated according to the optimized ground state. In addition, the free energy change Δ*G*_T1_ of electron transfer between dye‐A1 and additives was calculated and presented in Table 2.

**Scheme 3:**
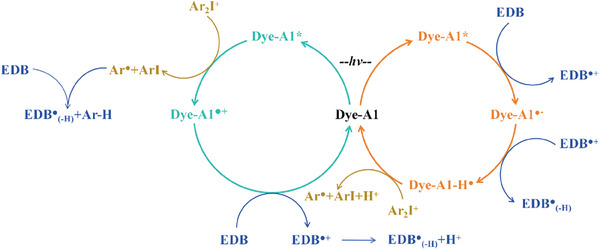
The photoinitiation mechanism of dye‐A1 as photoinitiator.

To sum up, the chemical reaction mechanism during photopolymerization is detailed in **Scheme** **3**. It mainly relies on the redox reaction between dye‐A1 and additives (EDB/Iod). In the first step, dye‐A1 was excited by light, transitioning from the ground state to the excited state (A1*). In the second step, A1* reacted with EDB and Iod to generate active free radicals that can initiate the polymerization. Specifically, A1* reacted with Iod to generate Ar**·** radical and A1**·** ^+^. Simultaneously, A1* reacted with EDB to generate EDB_(‐H)_ **·** free radical and dye‐H**·**. Last step, A1**·** ^+^ reacted with EDB and A1‐H**·** reacted with Ar_2_I^+^, and the electrons moved back to promote the regeneration of ground state dye‐A1, thus completing the catalytic cycle. It is worth noting that the cycle process of oxidation and reduction was demonstrated by the calculation results of frontier molecular orbital distribution and photochemical parameters.

#### 2.3.4 Free Radical Polymerization Kinetics under LED@405 nm and Sunlight

**Figure 5:**
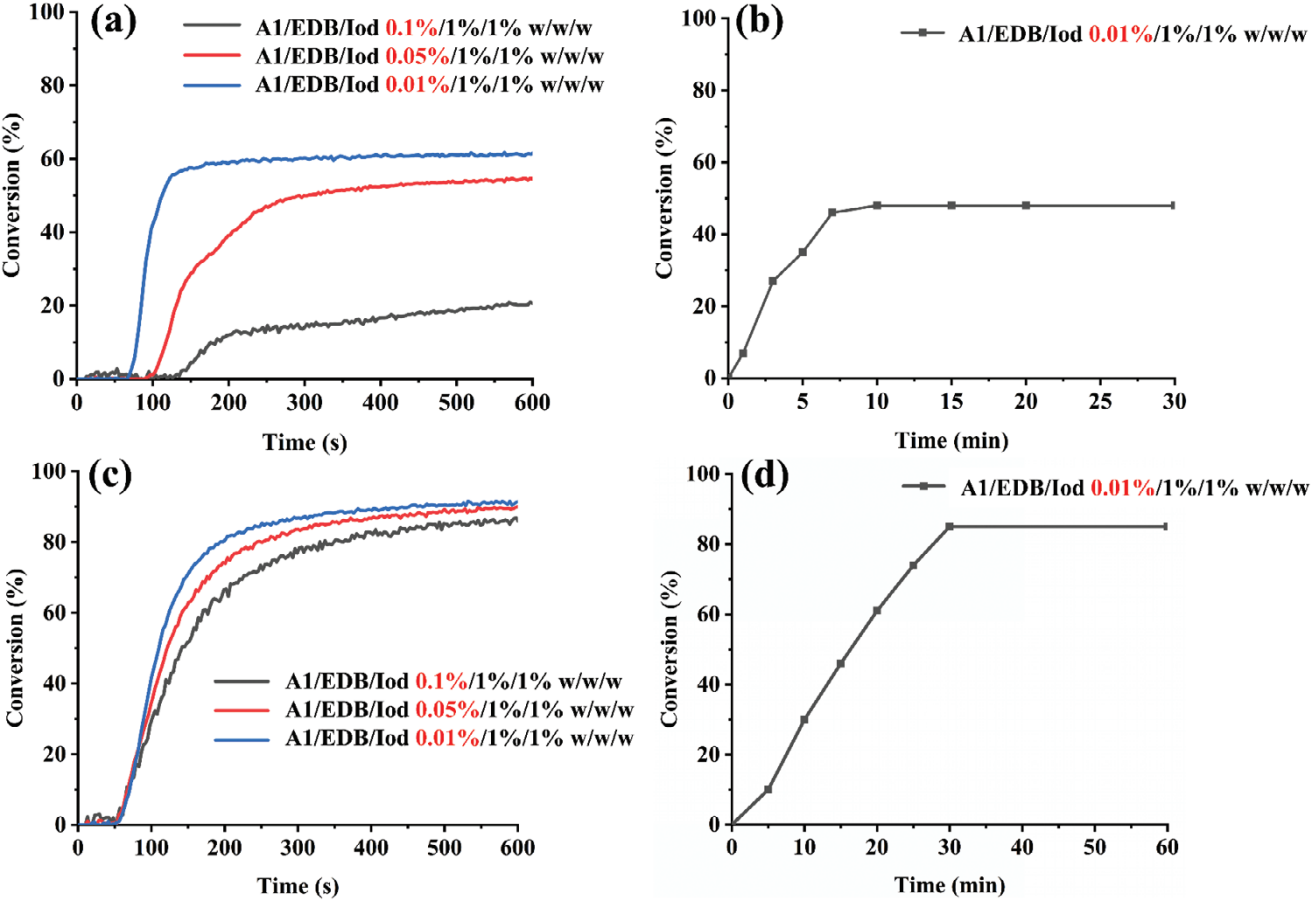
Photopolymerization profiles of the different formulations. Initiated by a) A1/EDB/Iod/TMPTA upon exposure to LED@405 nm. b) A1/EDB/Iod/TMPTA under sunlight at air. c) A1/EDB/Iod/PEGDA upon exposure to LED@405 nm. d) A1/EDB/Iod/PEGDA under sunlight at air. The thickness is 2 mm and the irradiation starts for *t* = 10 s.

To study the photoinitiation ability of dye‐A1, a three‐component system dye‐A1/EDB/Iod was designed to initiate the polymerization of monomers such as TMPTA and PEGDA with a LED@405 nm. The RT‐FTIR was used to monitor the conversions of acrylate double bond functional groups at room temperature to clarify the influence of the three‐component photoinitiating system on monomer polymerization kinetics. Specifically, the amount of the additives EDB/Iod was fixed to 1 wt% by controlling variables, and the effects of the three different contents of dye‐A1 (0.1 wt%, 0.05 wt%, and 0.01 wt%, relative to monomers) on the photopolymerization efficiency of TMPTA and PEGDA were studied. As shown in **Figure** **5**a,c, the polymerization profiles of TMPTA and PEGDA are respectively shown. It can be seen that by decreasing the dye concentrations, the conversions increased. When the concentration of dye‐A1 was 0.01 wt%, the monomer conversions was maximum, peaking at 60% with TMPTA and 90% with PEGDA. The high conversions achieved with the low concentration of dye A1 may be assigned to the solubility of dye in monomer. When the concentration of dye‐A1 was high, the deepening of the system color will lead to a poor light penetration, resulting in an internal filter effect due to the high light absorption property of dye. By reducing the dye concentrations, the formulation became clearer and more transparent, what is beneficial for the polymerization process.

The dye‐A1/EDB/Iod (0.01 wt%/1 wt%/1 wt%) system demonstrated the highest photoinitiation ability under LED@405 nm, so we also studied the ability of this formulation to initiate the polymerization under sunlight. Photopolymerization initiated under sunlight was carried out at 1–3 pm on February 28th, 2024 in Mulhouse, France. The polymerization of TMPTA and PEGDA under sunlight is shown in Figure 5b,c, respectively. Within 30 min, the conversion of TMPTA reached about 50%, while the conversion of PEGDA reached about 85%. The dye‐A1 showed an excellent photoinitiation ability in free radical polymerization.

### 2.4 Sunlight and UV–Visible Light Polymerized Hydrogels & 3D and 4D Printing Application

#### 2.4.1 Water Solubility of Dye‐A1 and Synthesis of Hydrogels Initiated by Dye‐A1

To study the dye‐A1 solubility in water, A1 was added to water gradually. As shown in Figure S7 (Supporting Information), A1 can be well dissolved in water in the concentration range of 5 wt% or less, which provided a basis for the subsequent synthesis of hydrogel.

**Figure 6:**
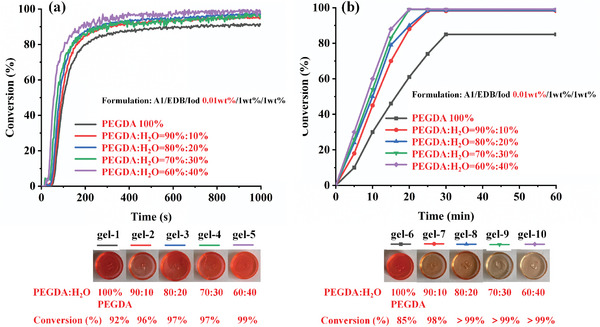
Photopolymerization profiles of hydrogels a) under LED@405 nm, b) under sunlight at air. The hydrogel formulations as follows: PEGDA 100 wt% (gel‐1), PEGDA: water 90 wt%:10 wt% (gel‐2), PEGDA:water 80 wt%:20 wt% (gel‐3), PEGDA:water 70 wt%:30 wt% (gel‐4), and PEGDA:water 60 wt%:40 wt% (gel‐5). Hydrogels synthesized under sunlight were as follows: PEGDA 100 wt% (gel‐6), PEGDA: water 90 wt%:10 wt% (gel‐7), PEGDA:water 80 wt%:20 wt% (gel‐8), PEGDA:water 70 wt%:30 wt% (gel‐9), and PEGDA: water 60 wt%:40 wt% (gel‐10). The thickness is 2 mm and the irradiation starts for *t* = 10 s.

From Section 2.3.4, the three‐component dye‐A1/EDB/Iod (0.01 wt%/1 wt%/1 wt%) system demonstrated the highest polymerization ability under both LED@405 nm and sunlight. Therefore, it was chosen for the preparation of hydrogels. As shown in Figure S8 (Supporting Information), when the water content was 0 wt%, 10 wt%, 20 wt%, 30 wt%, and 40 wt%, the precursor solution was clear, transparent, and stable. When the water content exceeded 40 wt%, white precipitates gradually separate out from the solution. This was because the additives EDB and Iod in the three‐component formulation were insoluble in water, and an excess of water can cause the additives to precipitate. Therefore, the five precursor solutions with water content of 0–40 wt% were selected to synthesize ten hydrogels (gel‐1 – 10) under visible light LED@405 nm and sunlight respectively, as shown in **Figure** **6**a,b. RT‐FTIR was used to investigate the conversions of precursor solution to hydrogels. Under the irradiation of LED@405 nm, the conversions of gel‐1– 5 were 92%, 96%, 97%, 97%, and 99%, respectively, while the conversions of gel‐6–10 were 85%, 98%, > 99%, > 99%, and > 99%, respectively under the sunlight. The photos of ten synthesized hydrogels are shown at the bottom of Figure 6. The results show that with the increase of the water content, the conversions of the precursor solution to hydrogels were higher.

#### 2.4.2 Water Swelling Properties

**Figure 7:**
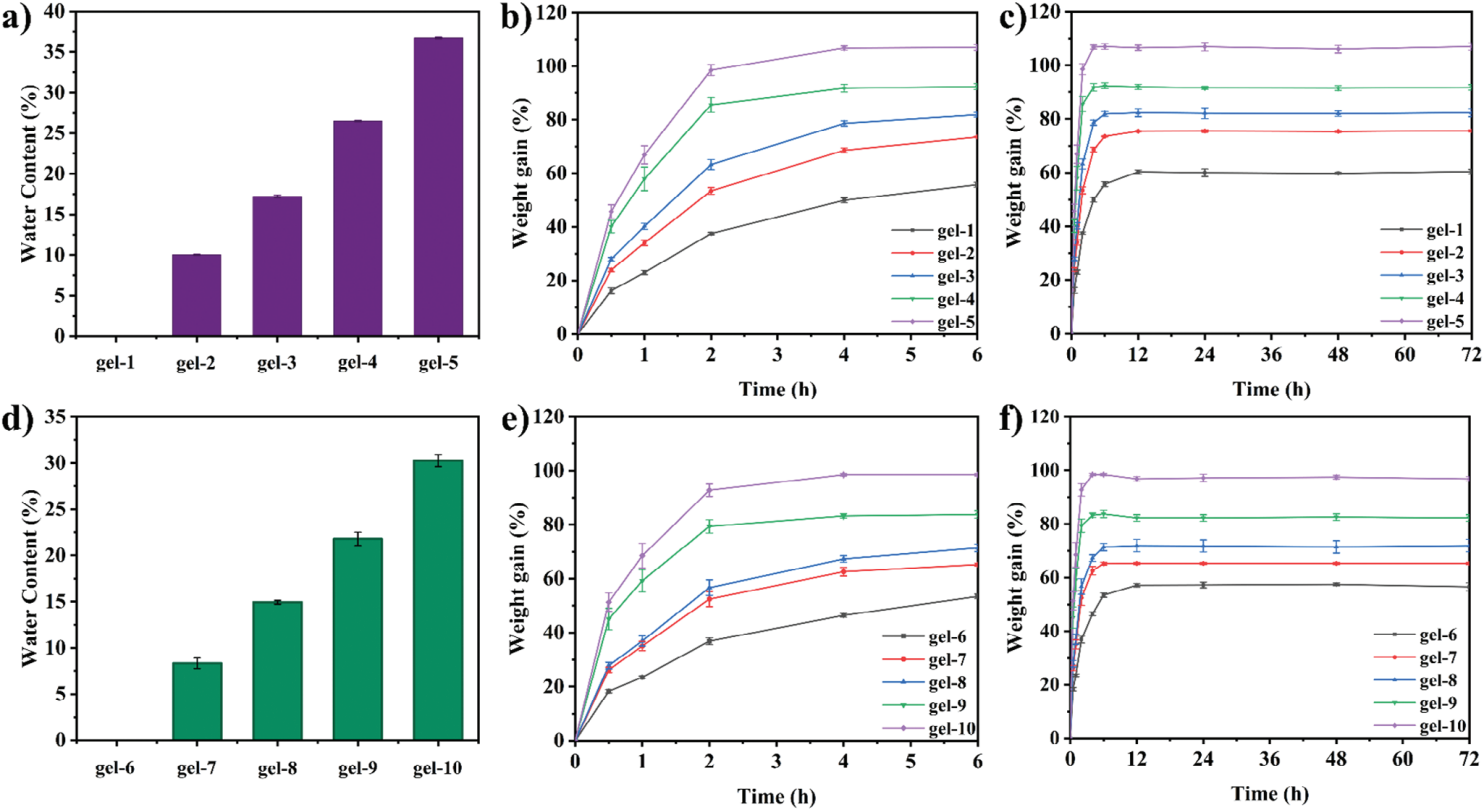
Water content of hydrogels a) under 405 nm. Water swelling of hydrogels b) within 6 h and c) within 72 h at RT. Water content of hydrogels d) under sunlight. Water swelling of hydrogels e) within 6 h and f) within 72 h at RT.

As shown in **Figure** **7**a,d, it can be seen that the water content of hydrogels synthesized under LED@405 nm was slightly higher than that synthesized under sunlight. The possible reason is that the hydrogels synthesized under LED@405 nm took a very short time, achieving conversions higher than 80% in only 200 s. In contrast, the hydrogels were polymerized within 30 min in sunlight, which increased the evaporation of water within the hydrogels.

As shown in Figure 7b,c, the swelling properties of gel‐1–5 are presented for 6 h and 72 h, respectively. Figure 7e,f shows the swelling properties of gel‐6–10 over the same time periods. All hydrogels reached the maximum swelling level within the first 6 h, and then remained in equilibrium. Interestingly, the higher the water content in the precursor formulation, the greater the swelling rate of the hydrogels. The possible reason is that the swelling rate of the hydrogels can change with the decrease of the number of hydrophilic groups.^[^ ^14^ ^]^ Reducing the content of PEGDA in the formulations and increasing the content of water can lead to the decrease of the swelling degree of the hydrogels. More interestingly, the water swelling percentage of the hydrogels synthesized at 405 nm are higher than that of the hydrogels synthesized in sunlight, and the water swelling rate of gel‐5 (PEGDA:water = 60 wt%:40 wt%) is more than 100%.

#### 2.4.3 Volume Swelling Properties of Hydrogels after Drying

**Figure 8:**
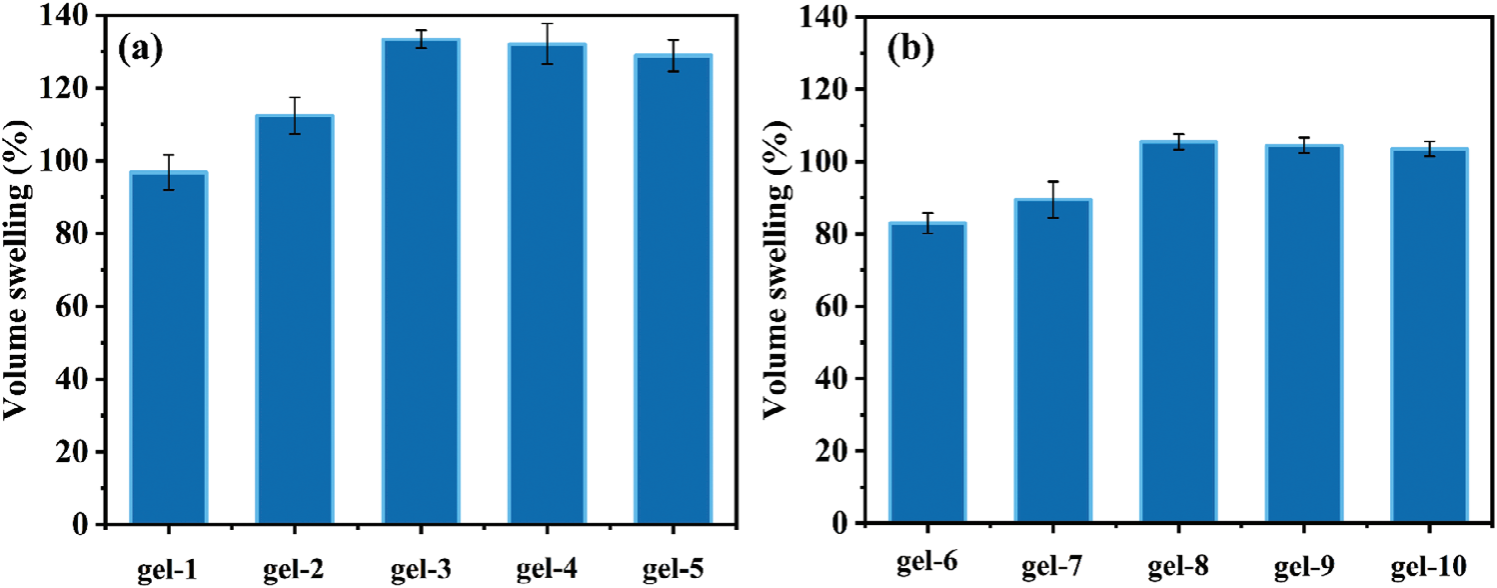
Volumetric swelling of hydrogels a) under LED@405 nm and b) under sunlight.

Hydrogels absorb water and swell at the same time, which is accompanied by the volume swelling. In order to observe the volume changes of hydrogels before and after water absorption, all hydrogels were made into cylinders with similar sizes, and then the volume swelling rates of ten hydrogels were calculated by accurately measuring the volumes of the cylinders after drying and swelling after water absorption reached a balance. As shown in **Figure** **8**a, it illustrates the volume swelling percentage of hydrogels synthesized under the irradiation of LED@405 nm. Figure 8b presents the volume swelling percentage of hydrogels synthesized under sunlight. It is very interesting to find that the volume swelling rate of hydrogels synthesized under LED@405 nm irradiation after drying is higher than that synthesized under sunlight after drying, and the gel‐3 (PEGDA:water = 80 wt%:20 wt%) synthesized at 405 nm shows the maximum volume swelling rate of 133% after drying.

#### 2.4.4 Application of 3D and 4D Printing Experiments

**Figure 9:**
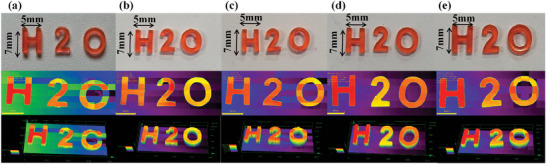
SEM and digital photos of 3D printing objects. All the formulations are A1/EDB/Iod 0.01 wt%/1 wt%/1 wt%. a) the monomers are PEGDA:100 wt%. b) The monomers are PEGDA:water 90 wt%:10 wt%. c) The monomers are PEGDA:water 80 wt%:20 wt%. d) The monomers are PEGDA:water 70 wt%:30 wt%. e) The monomers are PEGDA:water 60 wt%:40 wt%.

Due to the low or the absence of solubility of the current photoinitiating system in water, the application of hydrogels in the biological field is greatly limited.^[^ ^36^ ^]^ Based on the synthesized water‐soluble photoinitiator dye‐A1, the hydrogel fabricated from 3D printing with excellent water solubility have good potential biocompatibility. And the 3D printing of five hydrogels with different formulations (A1/EDB/Iod 0.01 wt%/1 wt%/1 wt%, the monomers are PEGDA:100 wt%, PEGDA:water 90 wt%:10 wt%, 80 wt%:20 wt%, 70 wt%:30 wt%, 60 wt%:40 wt%, respectively) in this study have been successfully studied. 3D pattern “H2O” could be obtained in all five hydrogels with high precision and clear outline in DLW experiment (see **Figure** **9**, the printed sizes are 7 mm in length, 5 mm in width and 2 mm in height). The morphology of the printed 3D pattern is observed by a digital camera and a numerical optical microscope. The 3D patterns with a smooth surface and an excellent spatial resolution were successfully manufactured in a short time.

**Figure 10:**
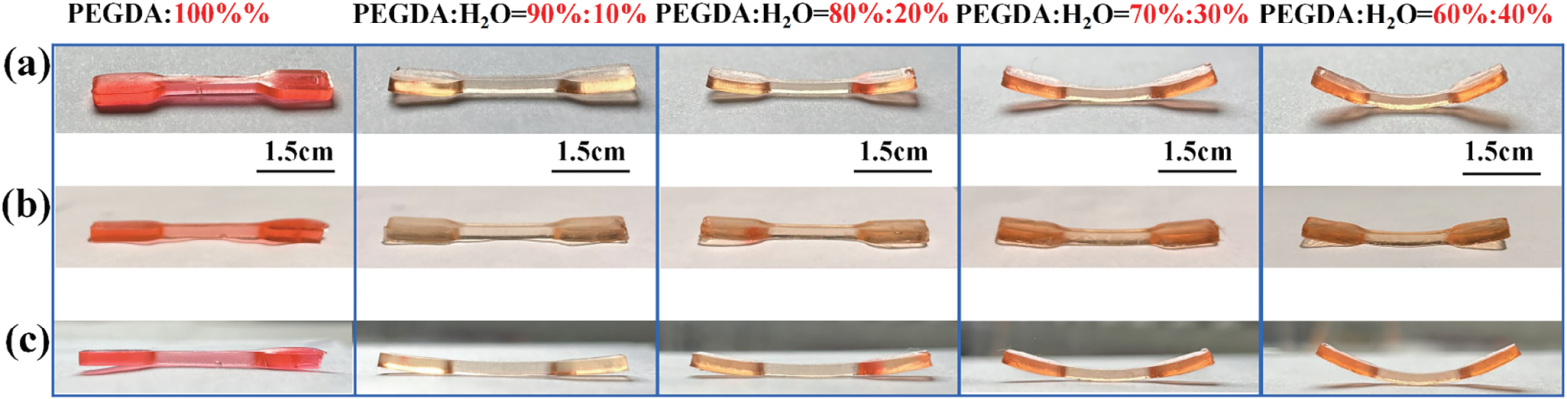
Deformation process of 4D printing products of five hydrogels based on water swelling and dehydration induced driving. a) After drying; b) After water swelling; c) After drying again.

In addition, five kinds of hydrogels were successfully manufactured into 4D printing products through the swelling and dehydration induced actuation of the 3D printed hydrogels. Specifically, as shown in **Figure** **10**, five hydrogels with different formulations were designed into dumbbell‐shaped objects with a length of 35 mm and a thickness of 2 mm. The hydrogels dumbbell just printed were flat, and after natural drying at room temperature (Figure 10a), it becomes bent due to dehydration. When the dried hydrogels were soaked in deionized water, the hydrogels dumbbell gradually changes back to the original flat state due to water swelling (Figure 10b). Subsequently, the expanded hydrogels dumbbell was taken out of the water and naturally dried, it was deformed back to a bent state due to dehydration (Figure 10c). These results show that the hydrogel prepared in this study exhibits a reversible deformation effect due to water responsiveness. It is particularly interesting to observe that with the increase of water content in the hydrogel formulations, the deformation degree of the printed hydrogels dumbbell was greater. According to this characteristic, the hydrogels can be designed, so that it can be transformed into different shapes under the stimulation of water environment. These swelling and contraction responses to water can be used to control the multiscale structural morphology of bone repair and support the potential of bone repair. It can appropriately encapsulate bone defects through swelling and shape memory effects. Additionally in the field of cancer therapy, 4D printing provides dynamic structures to address concerns about traditional resection and postoperative effects.^[^ ^37^ ^]^ In addition, this provides a potential material for the fabrication of bio‐soft robots, which are currently not popular in the medical field, since the hydrogel of this study has a good response to water and the resin used is biocompatible.^[^ ^38^, ^39^ ^]^ This study provides a reference for the use of hydrogel in biomedical applications.

## 3 Conclusion

In this study, we present a high‐performance photoinitiating system based on a new water‐soluble photoinitiator that can effectively initiate the polymerization of hydrogels under UV visible light and sunlight. The newly developed water‐soluble dyes exhibited an excellent photoinitiation ability, with a solubility in water exceeding 50 g L^−1^. Importantly, photoinitiating systems prepared with this dye could initiate the polymerization of hydrogels under sunlight without any additional aids. Furthermore, the hydrogel formulations developed could be used in 3D and even 4D printing. The shape memory functionality of hydrogels can allow for the creation of 3D structures with evolving characteristics, thereby expanding their applications in soft robotics and biomedicine.

## 4 Experimental Section

### Dye and Other Materials

The water‐soluble dye‐A1 was synthesized as detailed in the Supporting Information. The monomers, i.e., trimethylolpropane triacrylate (TMPTA) and polyethylene glycol diacrylate (PEGDA, average *M*n = 575 g mol^−1^) were purchased from Sartomer (France). Ethyl 4‐dimethylaminobenzoate (EDB) and *bis*(4‐*tert*‐butylphenyl)iodonium hexafluorophosphate (Iod) used as the additives of the photoinitiating systems were purchased from Lambson Ltd (United Kingdom). Chemical structures of all compounds are shown in Scheme 1.

### UV and Fluorescence Properties of Dye‐A1 (Including Steady‐State Photolysis, Fluorescence Quenching, and Fluorescence Lifetime)

The UV–visible absorption spectrum and steady‐state photolysis of dye‐A1 dissolved in methanol were investigated using the JASCO V730 spectrometer (the steady‐state photolysis used LED@405 nm UV–visible light to irradiate different systems, including dye‐A1 alone, dye‐A1/EDB, dye‐A1/Iod, and dye‐A1/EDB/Iod). The dye‐A1 concentration in methanol was kept at 2.5×10^−4^ m, the fluorescence spectrum was measured by JASCO FP‐6200 fluorescence spectrophotometer, in which singlet energy (*E*_S1_) was obtained from the intersection of normalized UV and fluorescence spectra. In addition, the fluorescence quenching experiment was carried out using the same instrument, and the electron transfer quantum yield (*ϕ*_et_) can be obtained from the fluorescence quenching curve by Equation (1). Finally, the lifetime of fluorescence excited state was measured using HORIBA PPD‐850 fluorometer.

(1) $$\phi_{et}=\frac{K_{\mathrm{sv}}\left[\mathrm{additive}\right]}{1+K_{\mathrm{sv}}\left[\mathrm{additive}\right]}$$

where, the Stern‐Volmer coefficients (*K_sv_*) correspond to the slopes of the Stern‐Volmer treatment in the fluorescence quenching experiments.

### Redox Potentials of Dye‐A1 Obtained by Cyclic Voltammetry

The redox potential of dye‐A1 was determined by dissolving a certain amount of dye‐A1 and tetrabutylammonium hexafluorophosphate in methanol under nitrogen atmosphere (cyclic voltammetry). The triplet energy level (*E*_T1_) determined by molecular modeling (Gaussian 03) and *E*_S1_ obtained as described in Section 2.2. According to Rehm‐Weller^[^ ^40^, ^41^ ^]^ equations, the change of free energy from singlet excited state (Δ*G* ^S1^_EDB_ or Δ*G* ^S1^_Iod_) and the triplet free energy (Δ*G* ^T1^_EDB_ or Δ*G* ^T1^_Iod_) in the electron transfer reaction between dye‐A1 and additives can be calculated. In particular, the *E*_ox_ of EDB is 1.0 V^[^ ^42^ ^]^ and the *E*_red_ of Iod is ‐0.7 V.^[^ ^43^ ^]^

### Free Radical Photopolymerization (FRP) under UV/Visible‐Light Irradiation and Sunlight‐Induced Polymerization under Air

In this study, the monomers used in photopolymerization were TMPTA and PEGDA, and the light sources were LED@405 nm and sunlight. The photoinitiating systems included one‐component (dye‐A1 alone), two‐component (dye‐A1/EDB and dye‐A1/Iod), and three‐component (dye‐A1/EDB/Iod) systems. First of all, the content of components in the formulations were investigated (since the one‐component, the two‐component formulations have poor photoinitiation ability, which were not discussed in this study). The photoinitiation ability of the three‐component dye‐A1/EDB/Iod systems in TMPTA under LED@405 nm were studied (including five different proportions of 1 wt%/1 wt%1 wt%, 0.5 wt%/1 wt%/1 wt%, 0.1 wt%/1 wt%/1 wt%, 0.05 wt%/1 wt%/1 wt%, and 0.01 wt%/1 wt%/1 wt%, relative to TMPTA). Finally, the formulation with the best photoinitiation ability was selected to initiate the polymerization of PEGDA under LED@405 nm and sunlight respectively. The characteristic peaks of acrylate functional groups were continuously detected using RT‐FTIR (JASCO FTIR‐6000) at about 6150 cm^−1^ (thick sample, about 2 mm). The final conversions of the functionally groups of the monomers were calculated using the following Equation:

(2) $$\mathrm{Conversion}\;\left(\%\right)=\left(1-\frac{At}{A0}\right)\times 100$$

where *A*_0_ is the initial peak area before light irradiation and *A*_t_ is the peak area after being irradiated with light for t s. The error bars on the final conversion are typically 1%.

In addition to LED@405 nm, the above formulations were directly induced by sunlight. Specific date was February 28th, 2024. The experiment site was located in Mulhouse, France (+77 43 ′ E, 47 75 ′ N), and the time is from 1 to 3 pm, where the weather conditions were sunny. The conversions of the acrylate functional groups were monitored using the RT‐FTIR spectroscopy.

### Solubility of Dye‐A1 in Water

The solubility of the synthesized initiator dye‐A1 in water was studied by adding it to deionized water and keeping stirring until reached its solubility limit. Solubility was calculated by the following Equation:

(3) $$\mathrm{Solubility}\;\left(\%\right)=\frac{m_{A1}}{m_{A1}+m_{\mathrm{water}}}\times 100$$

where, *m*_A1_ is the mass of dye‐A1, and *m*_water_ is the mass of water.^[^ ^44^ ^]^

### Cytotoxicity Test of Dye‐A1

Cell viability and proliferation assays were measured using C3H10 T1/2 cell line. The cells were seeded in six‐well plate on the day before the test, so as to be attached with 20 000 cells per well in Dulbecco's modified Eagle's medium (DMEM) 10% SVF at 5% CO_2_ and 37 °C with the concentration of 50 µm for each drug. The medium was replaced just before to start the acquisition with the concentration of 50 µm for each compound. Every 30 min, 10 images were acquired in each well, and the cell viability and proliferation were observed continuously for 20 h to test the toxicity of dye A1 to cells, and compared with the commercial initiator diphenyl(2,4,6‐trimethylbenzoyl)phosphine oxide (TPO) under the same conditions and concentrations.

### Synthesis of Hydrogels by LED@405 nm and Sunlight‐Induced Photopolymerization

The formulations of hydrogels contained the water‐soluble photoinitiator A1, additives EDB and Iod (A1/EDB/Iod 0.01 wt%/1 wt%/1 wt%, relative to PEGDA/water), monomer PEGDA, and deionized water. In this study, gel‐1 to gel‐10 hydrogels were successfully prepared upon exposure to the LED@405 nm or sunlight. Specifically, the hydrogels synthesized with the LED@405 nm were as follows: PEGDA 100 wt% (gel‐1), PEGDA: water 90 wt%:10 wt% (gel‐2), PEGDA: water 80 wt%:20 wt% (gel‐3), PEGDA: water 70 wt%:30 wt% (gel‐4), and PEGDA:water 60 wt%:40 wt% (gel‐5). Hydrogels synthesized under sunlight were as follows: PEGDA 100 wt% (gel‐6), PEGDA: water 90 wt%:10 wt% (gel‐7), PEGDA:water 80 wt%:20 wt% (gel‐8), PEGDA:water 70 wt%:30 wt% (gel‐9), and PEGDA:water 60 wt%:40 wt% (gel‐10).

### Water Content Determination of Hydrogels

The water contents of the prepared hydrogels were calculated by Equation (4), where *m*_0_ was the weight of the hydrogels, *m*_1_ was the weight after being naturally air‐dried at room temperature (25 °C) for 24 h or longer until no further change in weight could be detected. To ensure the accuracy of the experiment, the samples were synthesized in triplicate under the same conditions (N = 3).

(4) $$\mathrm{Water}\;\mathrm{content}\;\left(\%\right)=\frac{m_{0}-m_{1}}{m_{0}}\times 100$$

### Water Swelling Behavior

The water swelling characteristics of the polymer hydrogels were evaluated by the ratio of swollen‐weight in deionized water to the dry‐weight. The specific experimental procedure was as follows: the synthesized hydrogels were dried and accurately weighed. The dry hydrogels were then soaked in deionized water for various durations (0.5, 1, 2, 4, 6, 12, 24, 48, and 72 h) and accurately weighed until the equilibrium was attained. Prior to weighing, the water remaining on the surface of the hydrogel was gently dabbed off with a paper towel. The samples were synthesized in triplicate under the same conditions. The water swelling percentage of the hydrogel was calculated by the following Equation:

(5) $$\mathrm{Water}\;\mathrm{swelling}\;\mathrm{percentage}\;\left(\%\right)=\frac{W_{1}-W_{0}}{W_{0}}\times 100$$

where, *W*_1_ is the weight of the swollen hydrogels at equilibrium, *W*_0_ is the weight of the dry hydrogels.^[^ ^14^, ^45^ ^]^

### Volume Swelling Behavior

Hydrogels absorb water and expand in volume simultaneously. Ten hydrogels (gel‐1 to gel‐10) were made into cylindrical shapes to facilitate volume measurement (*V* = π*r ^2^*h). The hydrogels were naturally air‐dried at room temperature, and their initial volumes were recorded as *V*_0_. After that, the hydrogels were soaked in deionized water until their volume reached equilibrium, and it was recorded as *V*_1_. The samples were investigated in triplicate under the same conditions. The volumetric welling percentage of the hydrogels were calculated by the following Equation.

(6) $$\mathrm{Volumetric}\;\mathrm{swelling}\;\mathrm{percentage}\;\left(\%\right)=\frac{V_{1}-V_{0}}{V_{0}}\times 100$$

### Application of Hydrogels in 3D Printing

The 3D printing experiments of five different hydrogel formulations (PEGDA 100 wt% and PEGDA:water 90 wt%:10 wt%, 80 wt%:20 wt%, 70 wt%:30 wt%, 60 wt%:40 wt%) were carried out using the direct laser writing (DLW). In the DLW experiment, a computer‐programmed x, y stage (with a spot size of about 50 µm) and a 405 nm laser diode were used. The glass tank (length 3 cm, width 2 cm and height 0.3 cm) for printing was self‐made. Finally, the 3D printed patterns were observed using the digital optical microscope (DSX‐HRSU of Olympus).

### Preparation of Fabricated Hydrogels

Five hydrogel formulations (PEGDA 100 wt% and PEGDA:water 90 wt%:10 wt%, 80 wt%:20 wt%, 70 wt%:30 wt%, 60 wt%:40 wt%) prepared in advance were added to the mold and induced to polymerize in sunlight for 20 min. The resulting 3D polymer shape was a slender dumbbell, which was convenient for investigating its shape memory ability before and after water absorption and dehydration.

## Conflict of Interest

The authors declare no conflict of interest.

## Supporting information



Supporting Information

## Data Availability

The data that support the findings of this study are available from the corresponding author upon reasonable request.
