# Supplementary material for: High‐Performance Sunlight‐Induced Polymerized Hydrogels and Applications in 3D and 4D Printing
Source: Small. 2024 Dec 18;21(5):2411888. doi: 10.1002/smll.202411888 (PMC11798354; doi:10.1002/smll.202411888)

NANO · MICRO
small

Supporting Information

for *Small*, DOI 10.1002/smll.202411888

High-Performance Sunlight-Induced Polymerized Hydrogels and Applications in 3D and 4D Printing

Ji Feng, Zheng Liu, Tong Gao, Didier Gigmes, Fabrice Morlet-Savary, Michael Schmitt, Celine Dietlin, Tatiana Petithory, Laurent Pieuchot, Jing Zhang, Wenpeng Shan, Pu Xiao, Frédéric Dumur* and Jacques Lalevée**

Supporting Information

High-performance Sunlight-induced Polymerized Hydrogels and Application in 3D and 4D Printing

Ji Feng [a,b], Zheng Liu [c], Tong Gao [a,b], Didier Gigmes [c], Fabrice Morlet-Savary [a,b], Michael Schmitt [a,b], Celine Dietlin [a,b], Tatiana Petithory [a,b], Laurent Pieuchot [a,b], Jing Zhang [d], Wenpeng Shan [e], Pu Xiao [e]*, Frédéric Dumur [c]*, and Jacques Lalevée [a,b]*

[a] Université de Haute-Alsace, CNRS, IS2M UMR7361, F-68100 Mulhouse, France.

[b] Université de Strasbourg, France.

[c] Aix Marseille Univ, CNRS, ICR, UMR 7273, F-13397 Marseille, France.

[d] Future Industries Institute, University of South Australia, Mawson Lakes, SA 5095, Australia.

[e] State Key Laboratory of High-Performance Ceramics and Superfine Microstructure, Shanghai Institute of Ceramics, Chinese Academy of Sciences, Shanghai 200050, P. R. China.

E-mail address: jacques.lalevee@uha.fr (J. L.); frederic.dumur@univ-amu.fr (F.D.); p.xiao@mail.sic.ac.cn (P.X.)

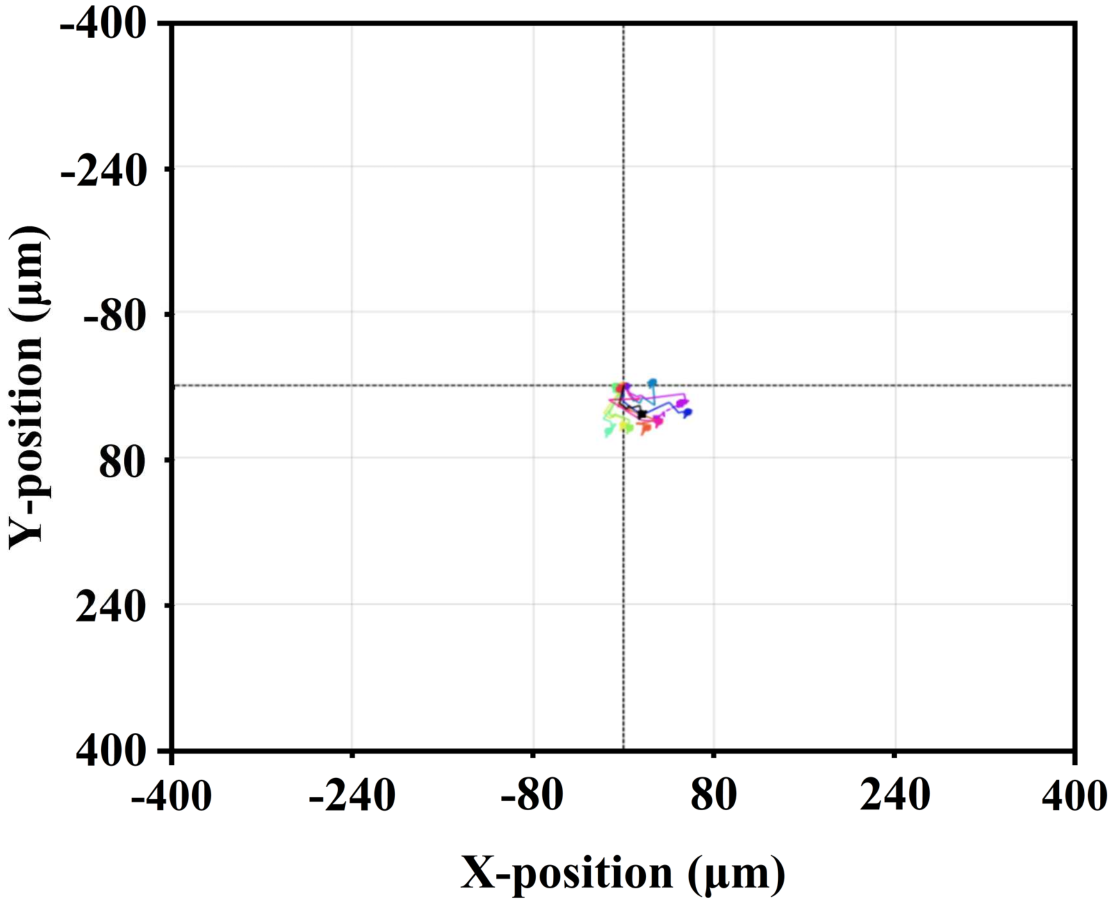

Figure S1. Single cell tracking of TPO.

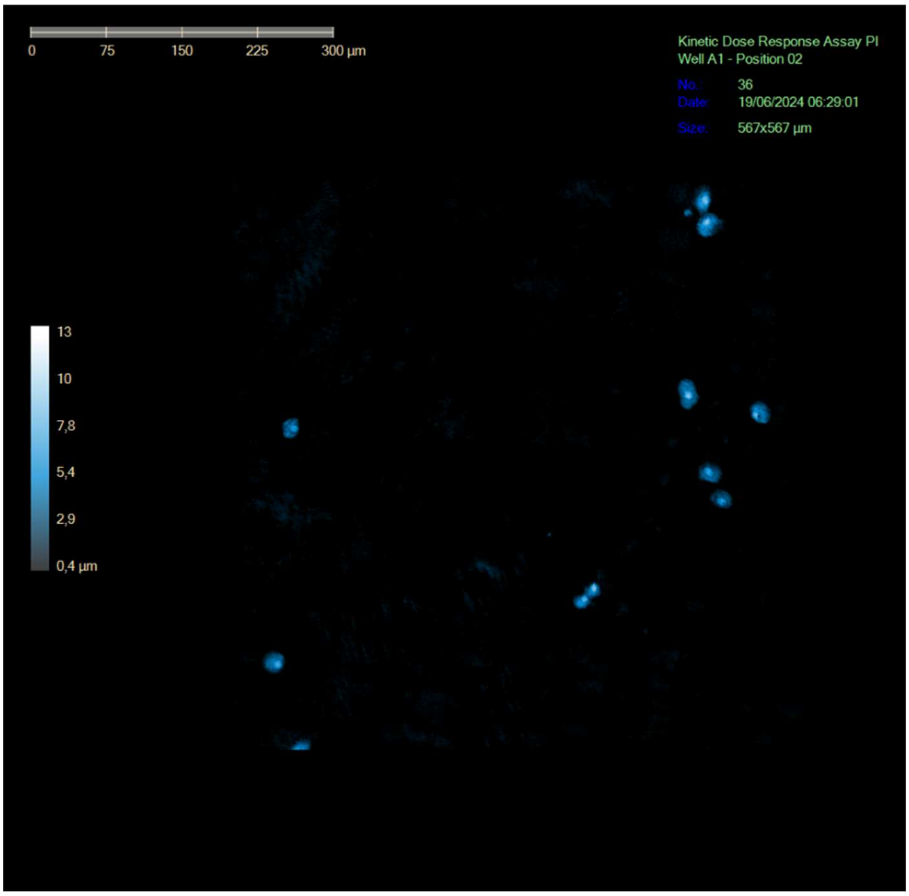

0
75
150
225
300 µm
Kinetic Dose Response Assay PI
Well A1 - Position 02
No.: 36
Date: 19/06/2024 06:29:01
Size: 567x567 µm
13
10
7,8
5,4
2,9
0,4 µm

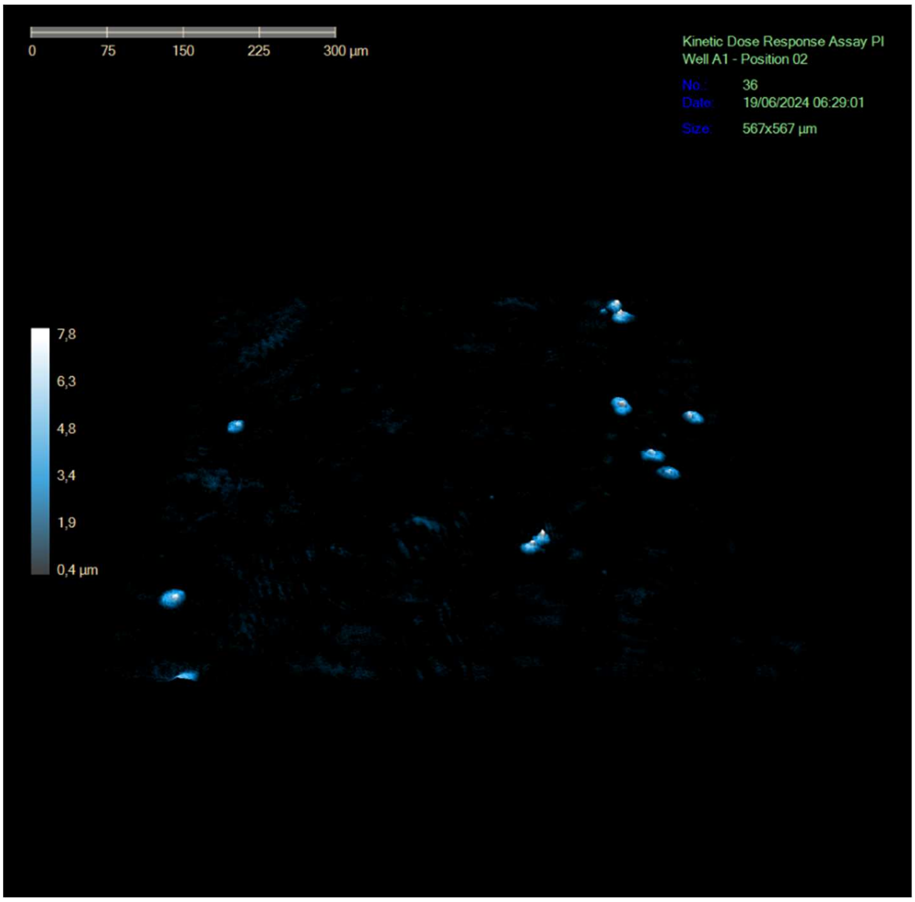

0
75
150
225
300 µm
Kinetic Dose Response Assay PI
Well A1 - Position 02
No.: 36
Date: 19/06/2024 06:29:01
Size: 567x567 µm
7,8
6,3
4,8
3,4
1,9
0,4 µm

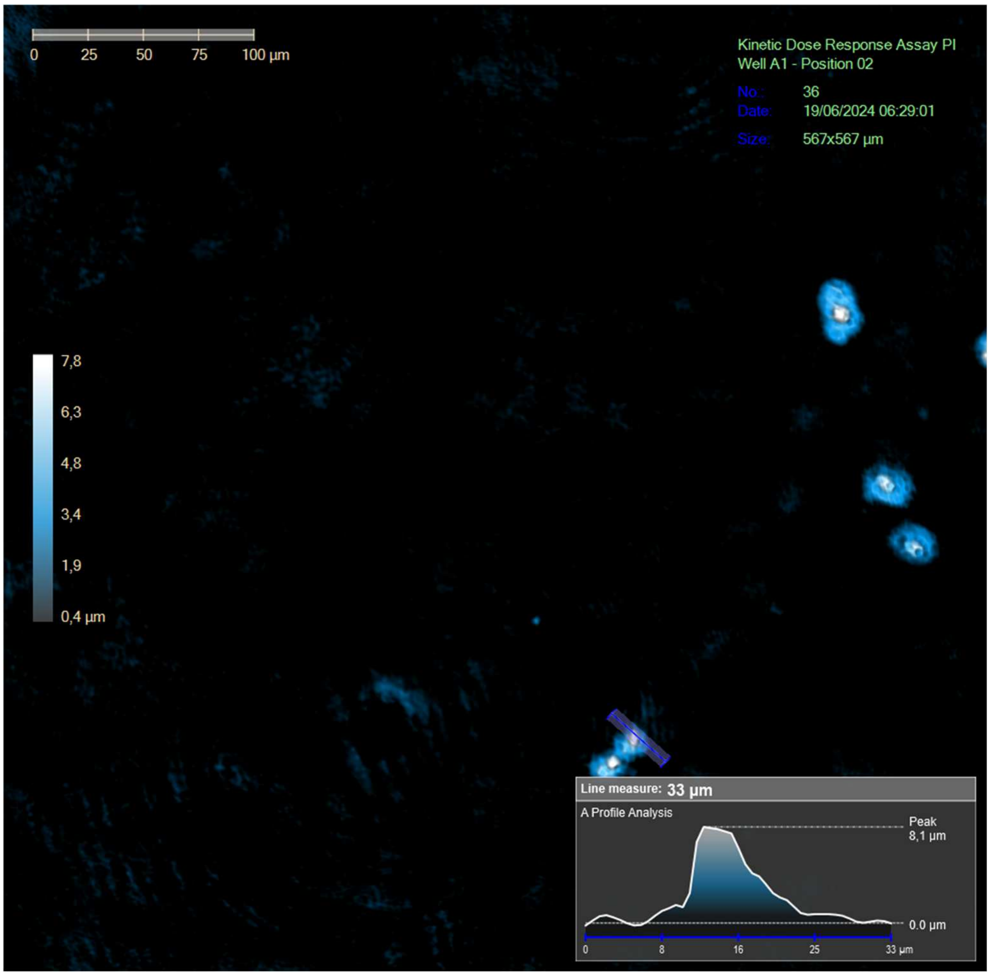

Figure S2. The Holomonitor images of TPO.

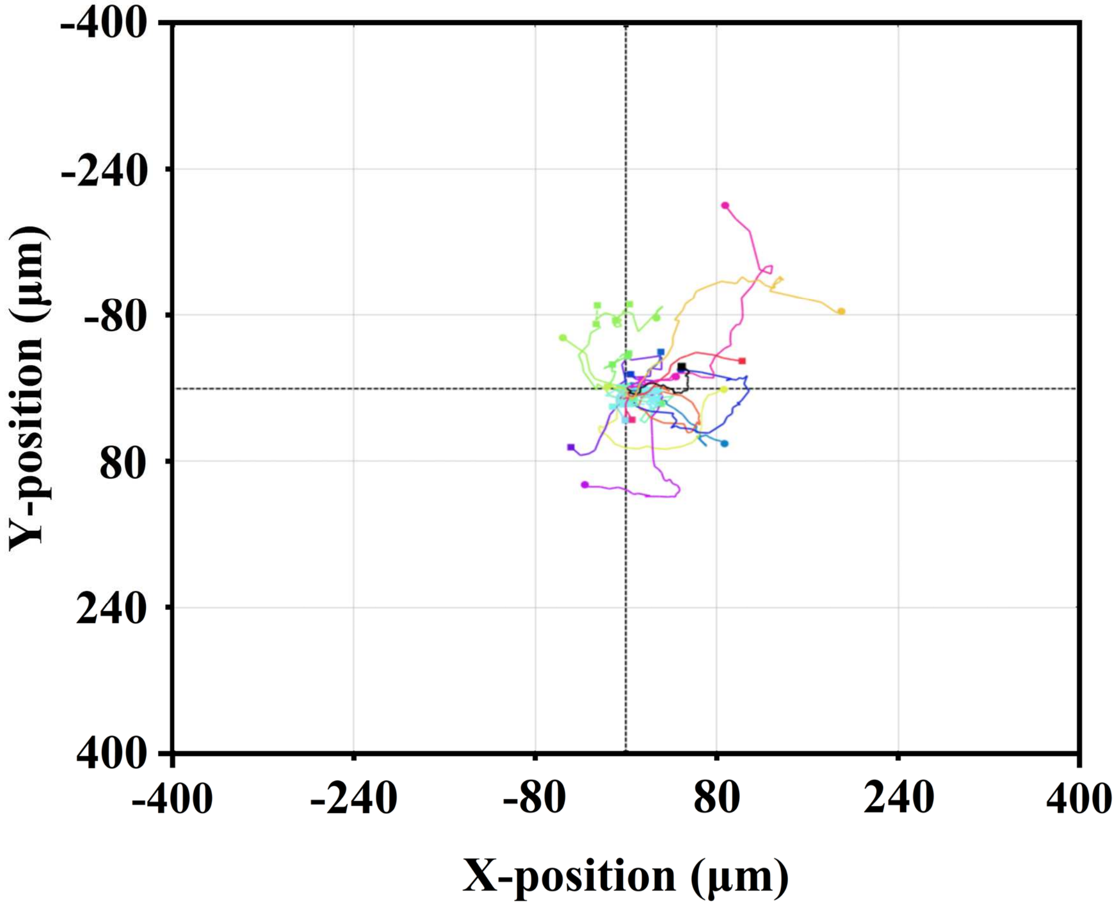

Figure S3. Single cell tracking of dye-A1.

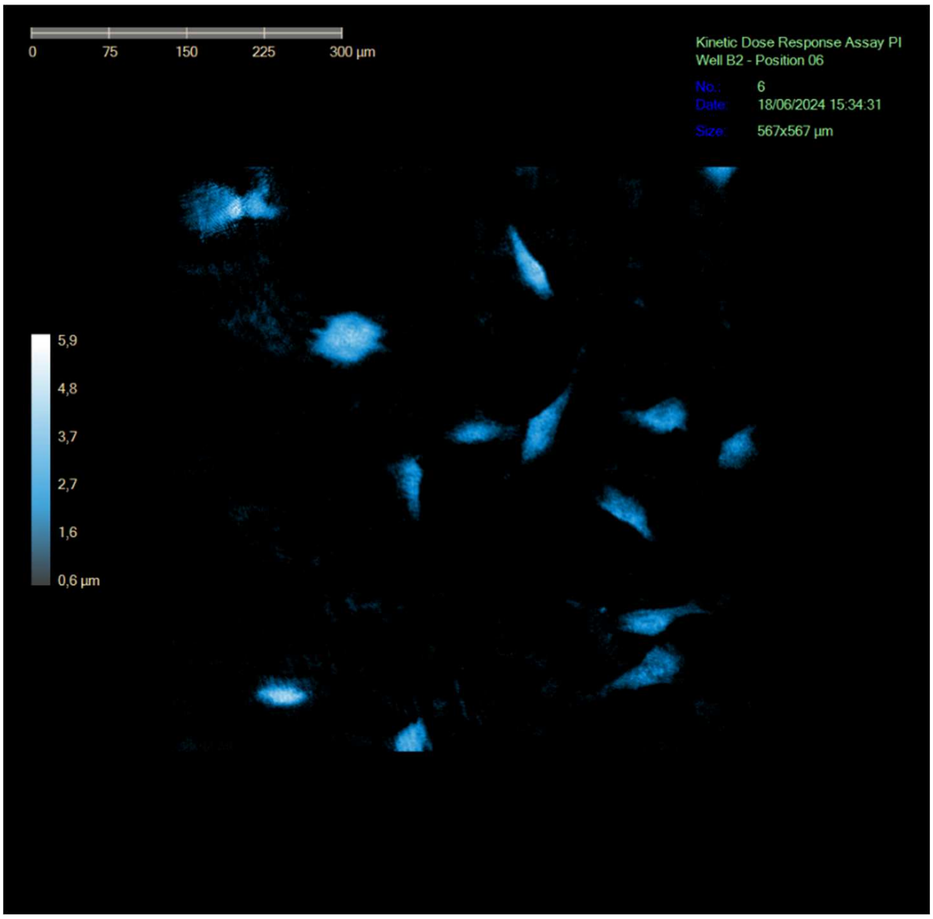
0
75
150
225
300 µm
Kinetic Dose Response Assay PI
Well B2 - Position 06
No.: 6
Date: 18/06/2024 15:34:31
Size: 567x567 µm
5,9
4,8
3,7
2,7
1,6
0,6 µm

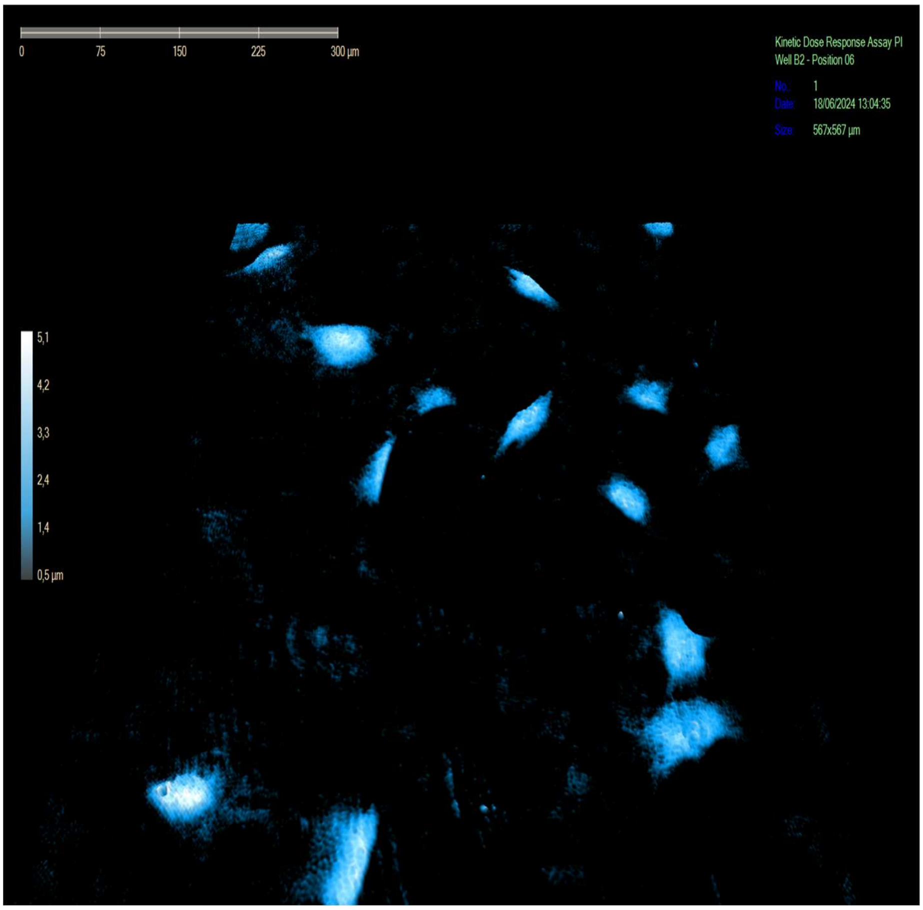
0
75
150
225
300 µm
Kinetic Dose Response Assay PI
Well B2 - Position 06
No.: 1
Date: 18/06/2024 13:04:35
Size: 567x567 µm
5,1
4,2
3,3
2,4
1,4
0,5 µm

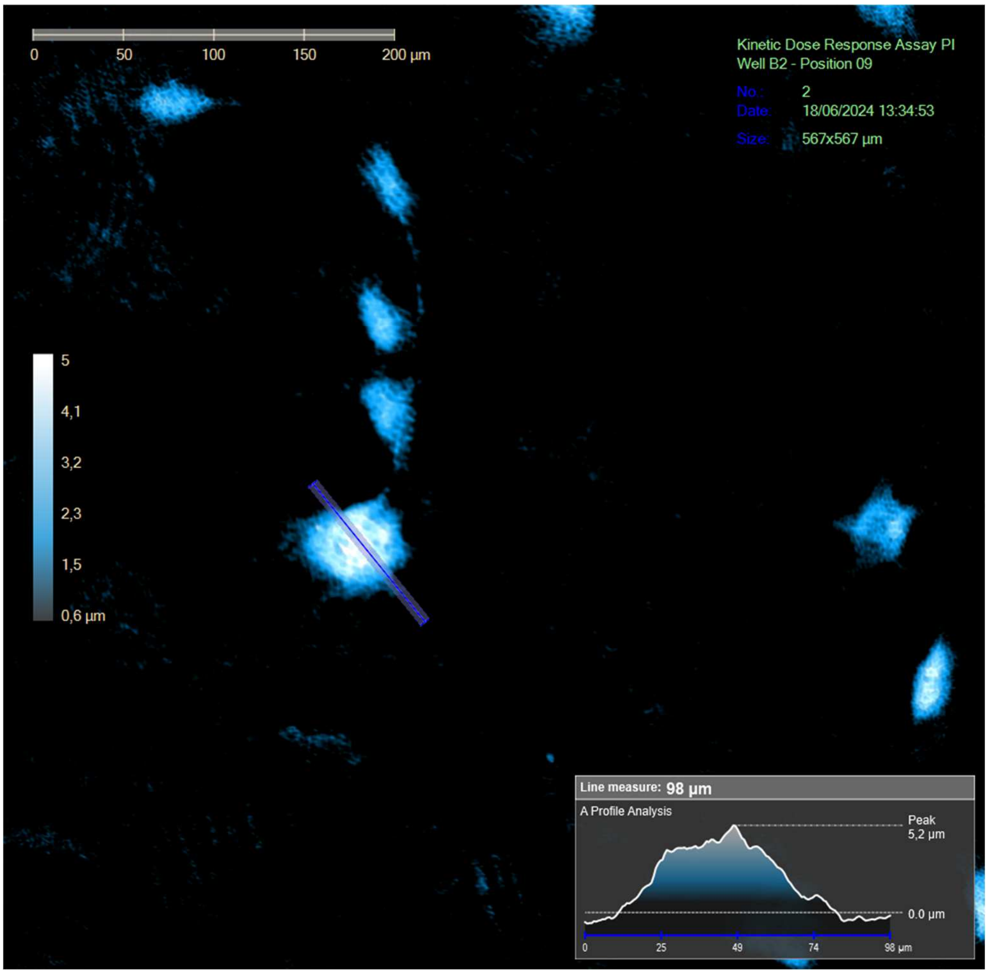

Figure S4. The Holomonitor images of dye-A1.

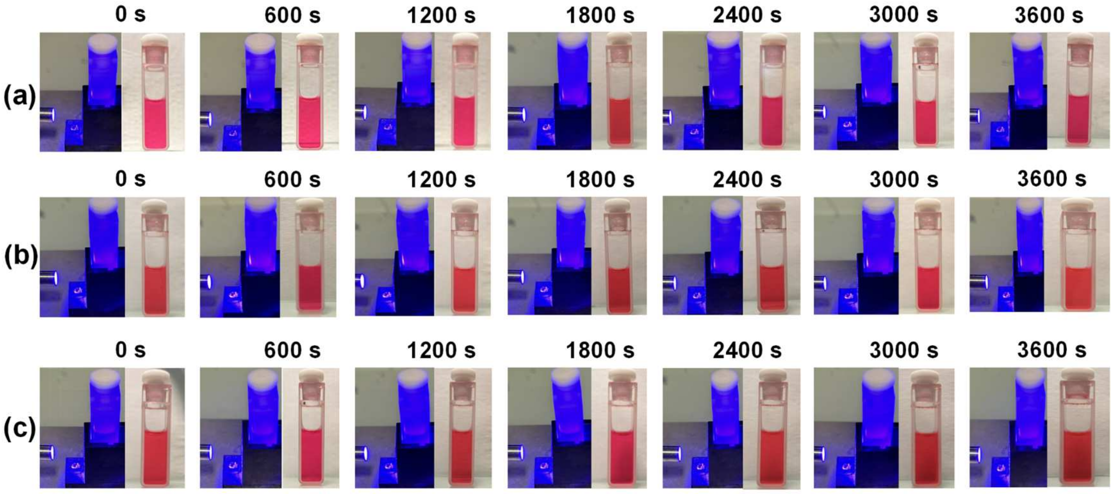

Figure S5. The changes during photolysis (a) A1/EDB; (b) A1/Iod; (c) A1/EDB/Iod.

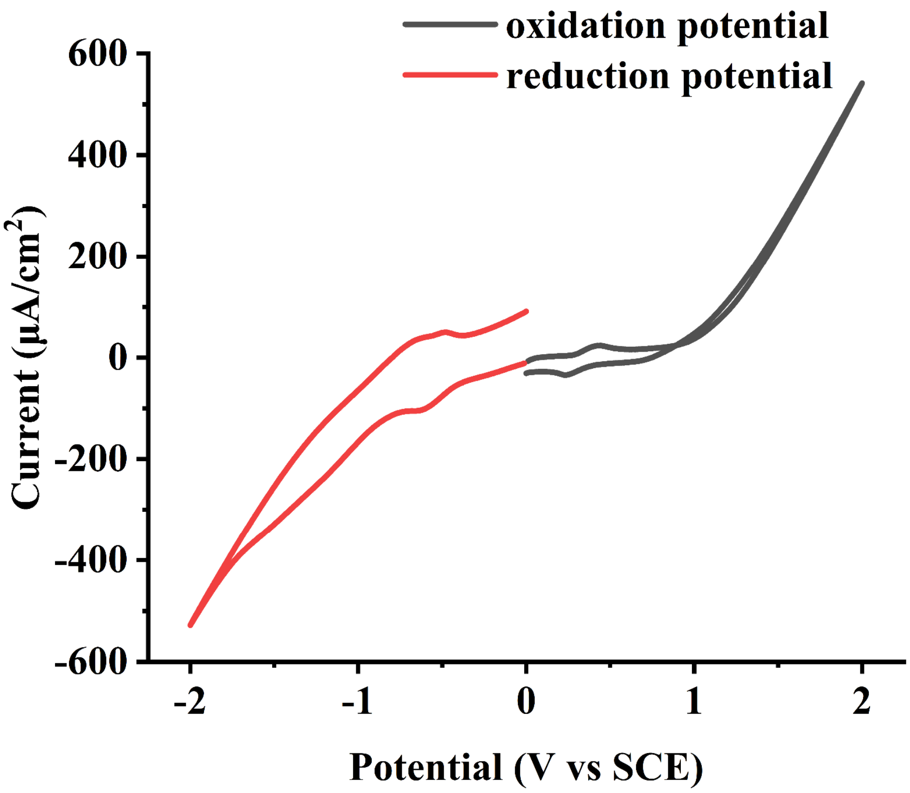

Figure S6. Cyclic voltammetry of electrochemical reactions of the dye-A1 in methanol solvent against saturated calomel electrode (SCE) under N_2 saturated solution.

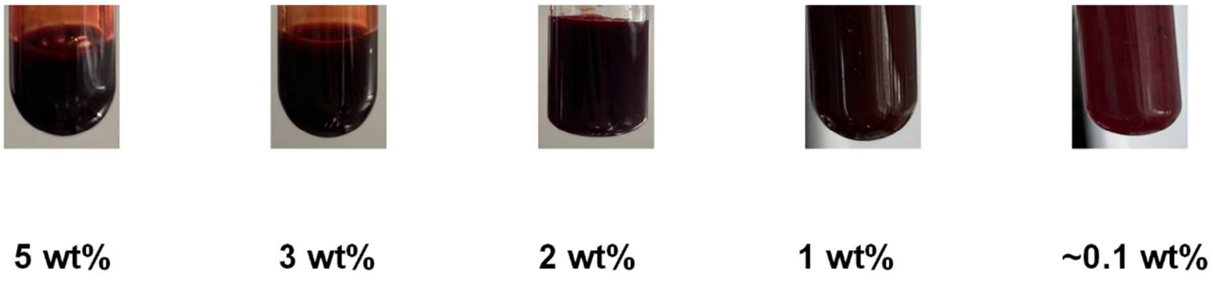

Figure S7. Solubility of dye-A1 in water.

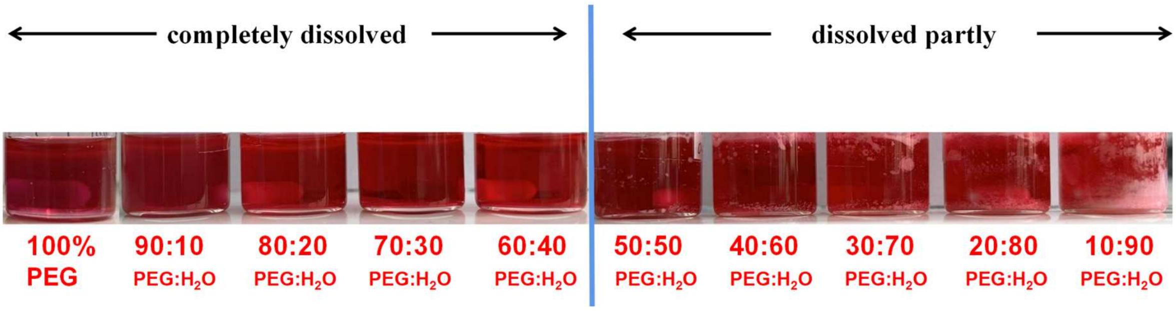

Figure S8. The dissolution of different formulations.

General information

All reagents and solvents were purchased from Aldrich or Alfa Aesar and used as received without further purification. Mass spectroscopy was performed by the Spectropole of Aix-Marseille University. ESI mass spectral analyses were recorded with a 3200 QTRAP (Applied Biosystems SCIEX) mass spectrometer. The HRMS mass spectral analysis was performed with a QStar Elite (Applied Biosystems SCIEX) mass spectrometer. Elemental analyses were recorded with a Thermo Finnigan EA 1112 elemental analysis apparatus driven by the Eager 300 software. ^{1}H and ^{13}C NMR spectra were determined at room temperature in 5 mm o.d. tubes on a Bruker Avance 400 or a Bruker Avance 300 spectrometer of the Spectropole: ^{1}H (400 MHz), ^{1}H (300 MHz), ^{13}C (100 MHz), and ^{13}C (75 MHz). All ^{1}H chemical shifts were referenced to the solvent peak $CDCl_3$ (7.26 ppm), DMSO-d_6 (2.49 ppm), D_2O (4.79 ppm), the ^{13}C chemical shifts were referenced to the solvent peak $CDCl_3$ (77.0 ppm) and D_2O (no signal).

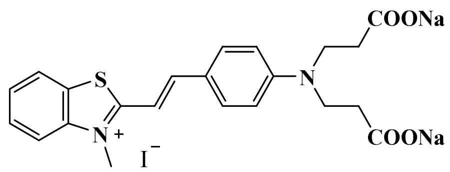

Figure S3. Chemical structure of the water-soluble dye-A1 investigated in this work.

Synthesis of dimethyl 3,3'-(phenylazanediyl)dipropionate

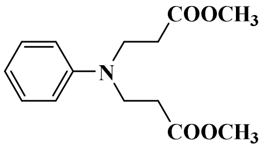

Chemical Formula: $C_{14}H_{19}NO_4$
Molecular Weight: 265,3090

Aniline (0.98 mL, 10.74 mmol, M = 93.13 g/mol, d = 1.02 g/mL), methyl acrylate (2.92 mL, 32.21 mmol, M = 86.09 g/mol, d = 0.95 g/mL), acetic acid (1.23 mL, 21.48 mmol, M = 60.05 g/mol, d = 1.05 g/mL) and *p*-benzenediol (0.04 g, 0.32 mmol, M = 110.11 g/mol) were added into a 100 mL flask and the solution was refluxed overnight at 102°C. After cooling to room temperature, excess of methyl acrylate and acetic acid were removed by rotary evaporator, and the resulting liquid was used without further purification (2.76 g, 97.0% yield).

^{1}H NMR (400 MHz, $CDCl_3$) δ 7.14 (dd, *J* = 8.7, 7.3 Hz, 2H), 6.66 – 6.60 (m, 3H), 3.60 – 3.54 (m, 10H), 2.53 – 2.48 (t, *J* = 7.2 Hz, 4H).

^{13}C NMR (101 MHz, $CDCl_3$) δ 172.57 (s), 146.68 (s), 129.48 (s), 117.12 (s), 112.66 (s), 51.70 (s), 46.89 (s), 32.23 (s).

^{1}H NMR spectrum of dimethyl 3,3'-(phenylazanediyl)dipropionate

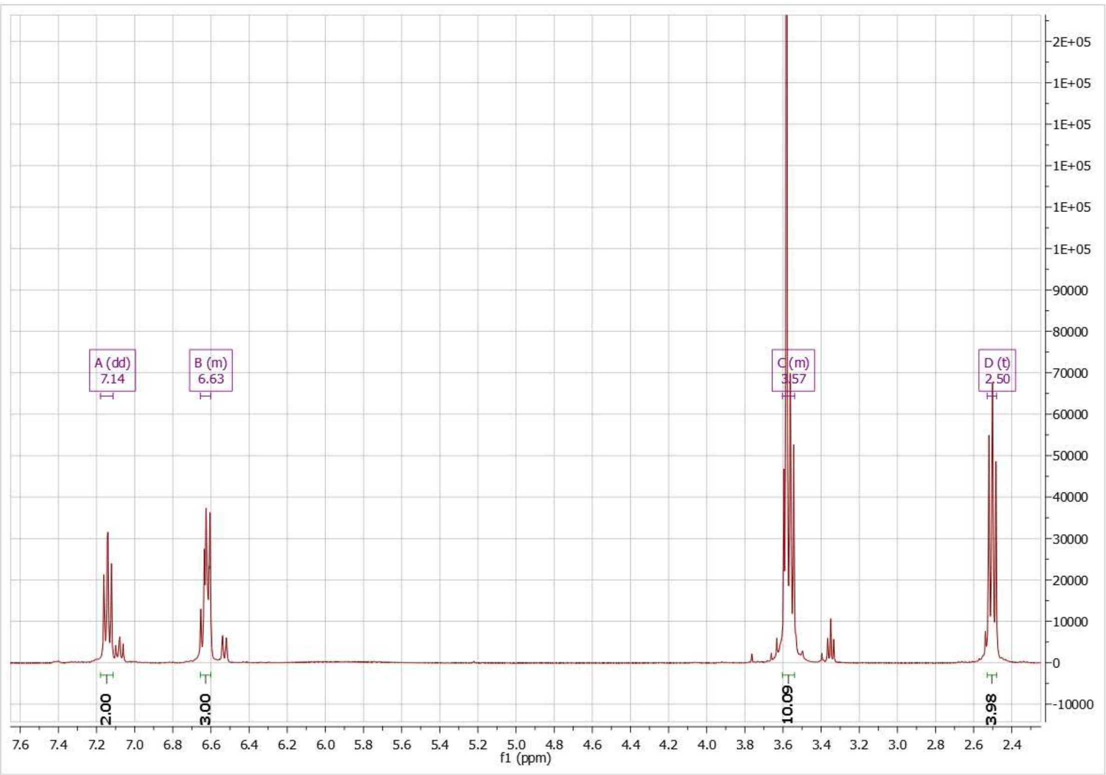

^{13}C NMR spectrum of dimethyl 3,3'-(phenylazanediyl)dipropionate

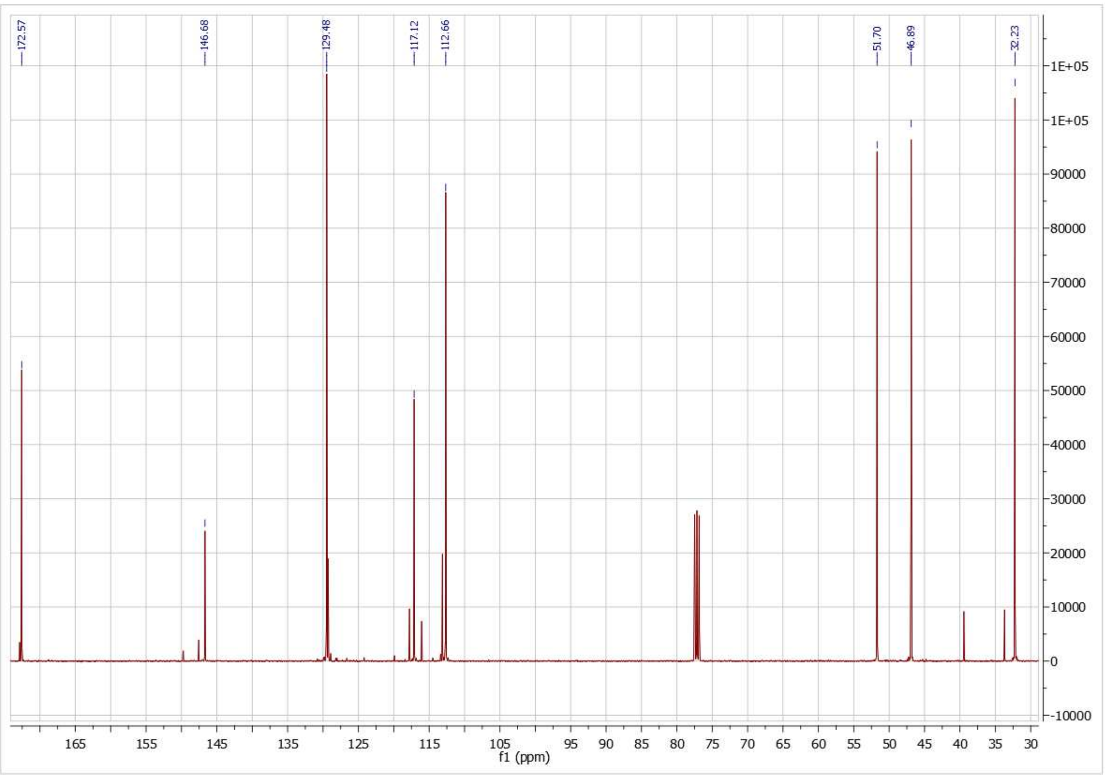

Synthesis of dimethyl 3,3'-((4-formylphenyl)azanediyl)dipropionate

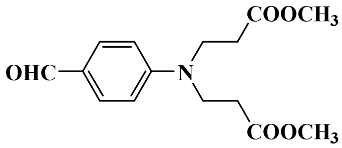

Chemical Formula: $C_{15}H_{19}NO_5$
Molecular Weight: 293,3190

Phosphorus pentachloride (3.14 g, 15.08 mmol, M = 208.22 g/mol) was added slowly to DMF (100 mL). After stirring at 0 °C and room temperature for 1 h respectively, 3,3'-(phenylazanediyl)dipropionate (2.00 g, 7.54 mmol, M = 265.31 g/mol) was slowly added. After stirring at 75°C overnight and then cooled to room temperature, the solution was poured in ice water and neutralized with 2.5% M KOH. The solution was subsequently washed with DCM and dried over $MgSO_4$. After evaporation of the volatiles, the pure liquid was used without any further purification (2.13 g, 96.4% yield).

^{1}H NMR (300 MHz, $CDCl_3$) δ 9.75 (s, 1H), 7.74 (d, *J* = 8.9 Hz, 2H), 6.71 (d, *J* = 8.9 Hz, 2H), 3.76 (t, *J* = 7.2 Hz, 4H), 3.69 (s, 6H), 2.63 (t, *J* = 7.2 Hz, 4H).

^{13}C NMR (101 MHz, $CDCl_3$) δ 190.04 (s), 171.77 (s), 151.26 (s), 132.12 (s), 125.88 (s), 111.15 (s), 51.79 (s), 46.56 (s), 31.90 (s).

The synthesis process and analyses are consistent with those previously reported in the literature [Xing Huang, Xiaopu Wang, Yuxia Zhao. Study on a series of water-soluble photoinitiators for fabrication of 3D hydrogels by two-photon polymerization[J]. Dyes and Pigments, 2017, 141: 413-419.]

^{1}H NMR spectrum of dimethyl 3,3'-((4-formylphenyl)azanediyl)dipropionate

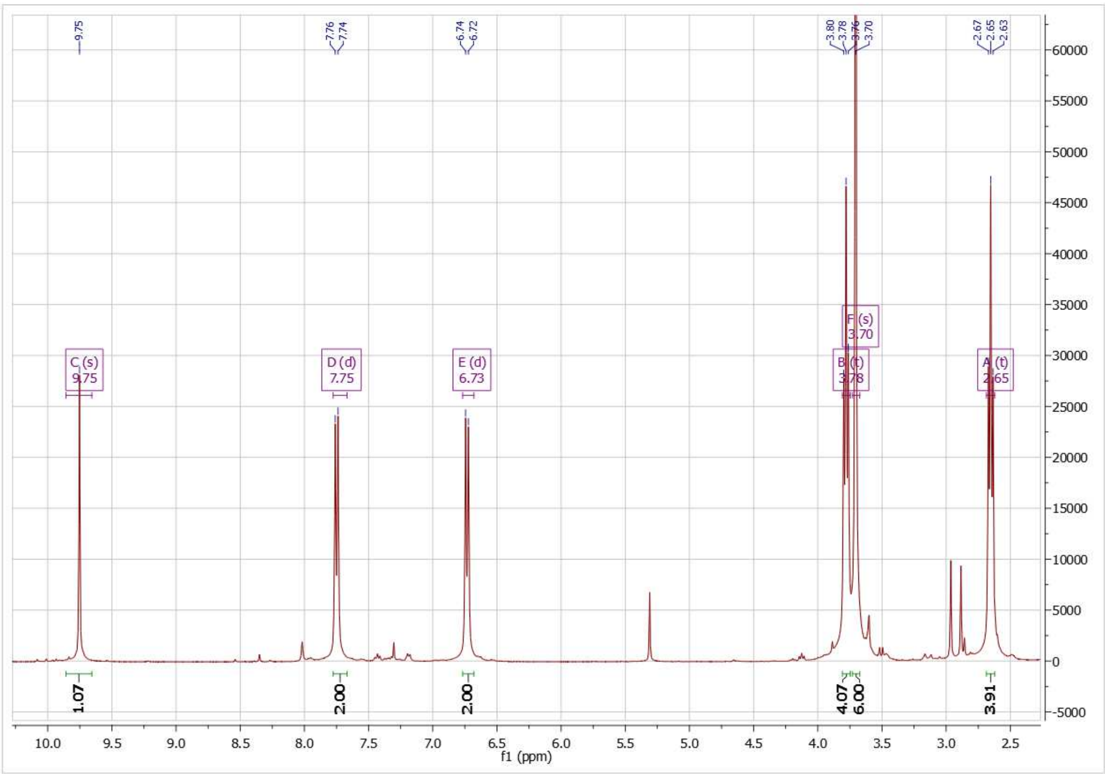

^{13}C NMR spectrum of dimethyl 3,3'-((4-formylphenyl)azanediyl)dipropionate

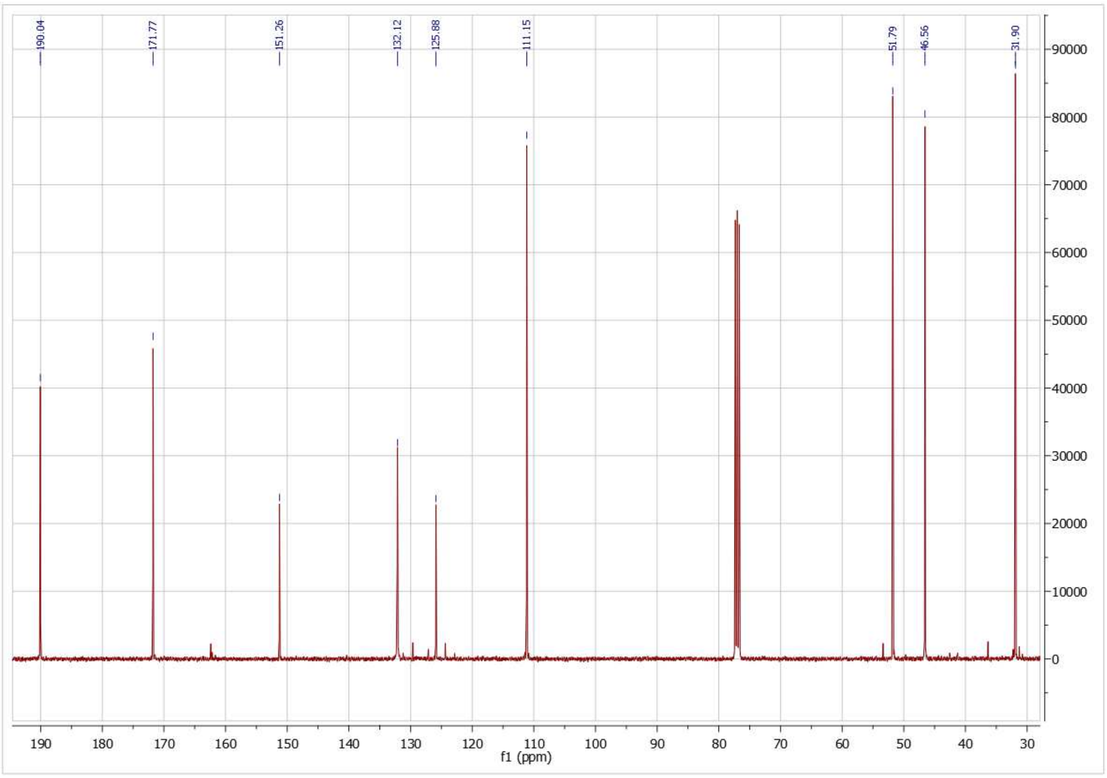

Synthesis of sodium 3,3'-((4-formylphenyl)azanediyl)dipropionate

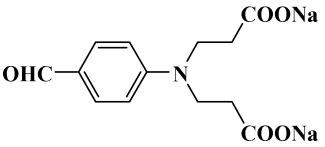

Chemical Formula: $C_{13}H_{13}NNa_2O_5$
Molecular Weight: 309,2285

3,3'-((4-Formylphenyl)azanediyl)dipropionate (2.00 g, 6.82 mmol, M = 293.32 g/mol) and NaOH (0.55 g, 13.64 mmol, M = 40 g/mol) were added to water (350 mL) and the resulting solution was stirred at room temperature overnight. After most of water was removed by rotary evaporation, the red solid compound (1.23 g, 58.3% yield) was recrystallized from ethanol.

1H NMR (400 MHz, D_2O) δ 9.41 (s, 1H), 7.70 (d, *J* = 8.8 Hz, 2H), 6.81 (d, *J* = 8.9 Hz, 2H), 3.70 – 3.65 (t, *J* = 7.2 Hz, 4H), 2.49 – 2.44 (t, *J* = 7.2 Hz, 4H).

^{13}C NMR (101 MHz, D_2O) δ 193.43 (s), 180.60 (s), 153.19 (s), 123.37 (s), 111.36 (s), 57.43 (s), 47.64 (s), 35.14 (s), 16.83 (s).

^{1}H NMR spectrum of sodium 3,3'-((4-formylphenyl)azanediyl)dipropionate

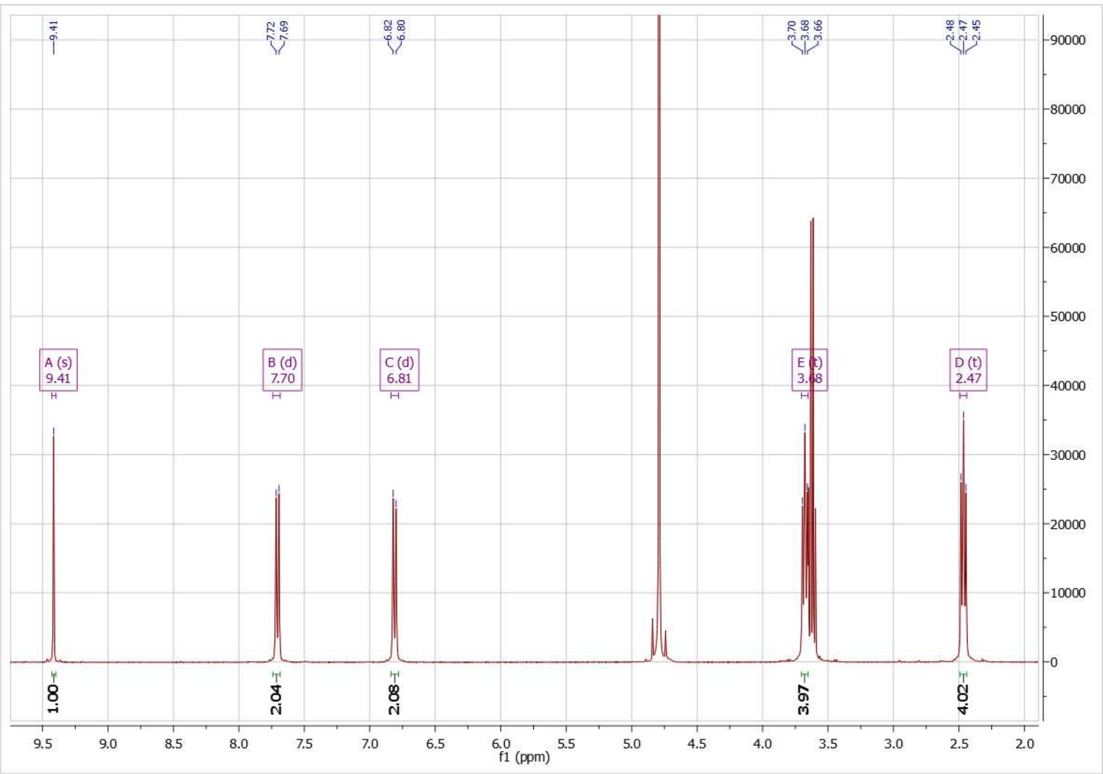

^{13}C NMR spectrum of sodium 3,3'-((4-formylphenyl)azanediyl)dipropionate

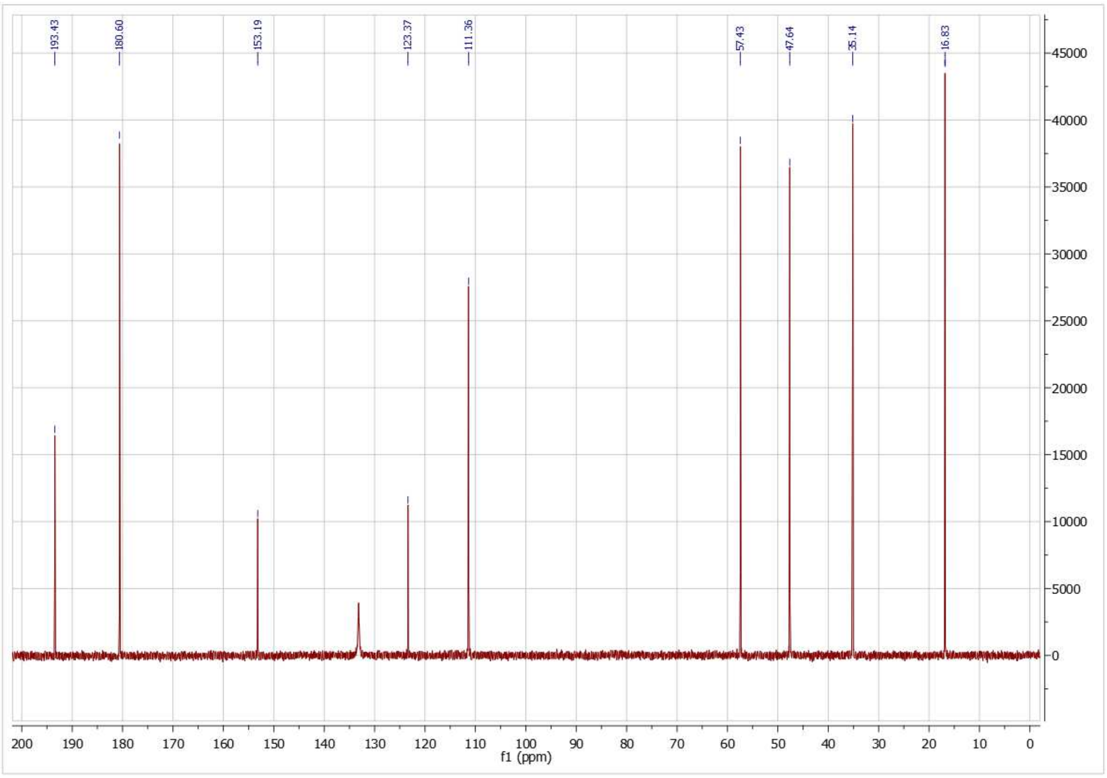

Synthesis of 2,3-dimethylbenzo[*d*]thiazol-3-ium iodide

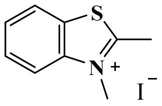

Chemical Formula: $C_9H_{10}INS$
Molecular Weight: 291,1505

2-Methylbenzo[*d*]thiazole (2.00 g, 13.40 mmol, M = 149.21 g/mol) and iodomethane (2.00 g, 14.07 mmol, M = 141.94 g/mol) were added to DMF (10 mL) and the solution was stirred at 60°C overnight. After cooling to room temperature, ethyl acetate was added to the solution and the solution was filtered to collect the precipitate. After recrystallizing three times using ethyl acetate, the pure product was obtained and used without any further purification (3.74 g, 95.8% yield).

^{1}H NMR (400 MHz, DMSO-d_6) δ 8.46 (d, *J* = 8.0 Hz, 1H), 8.29 (d, *J* = 8.4 Hz, 1H), 7.88 (t, *J* = 7.3 Hz, 1H), 7.79 (t, *J* = 7.7 Hz, 1H), 4.21 (s, 3H), 3.19 (s, 3H).

^{13}C NMR (101 MHz, DMSO-d_6) δ 177.59 (s), 141.99 (s), 129.66 (s), 129.10 (s), 128.46 (s), 124.95 (s), 117.20 (s), 36.84 (s), 17.83 (s).

^{1}H NMR spectrum of 2,3-dimethylbenzo[*d*]thiazol-3-ium iodide

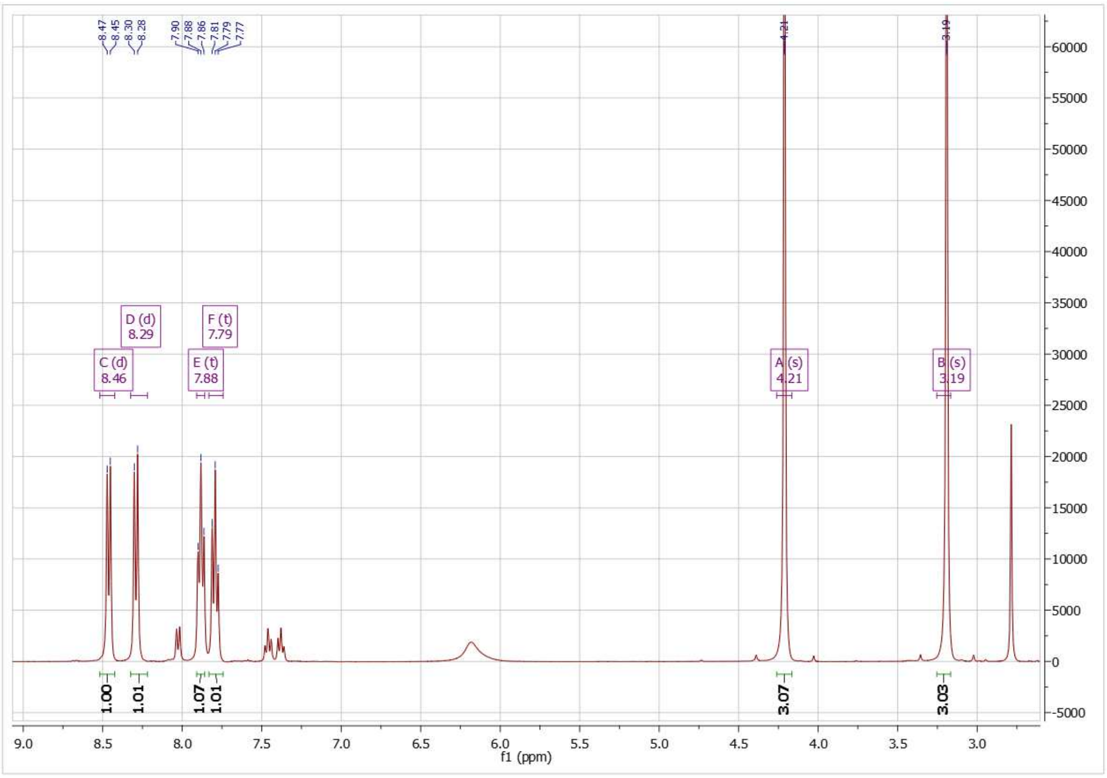

^{13}C NMR spectrum of 2,3-dimethylbenzo[*d*]thiazol-3-ium iodide

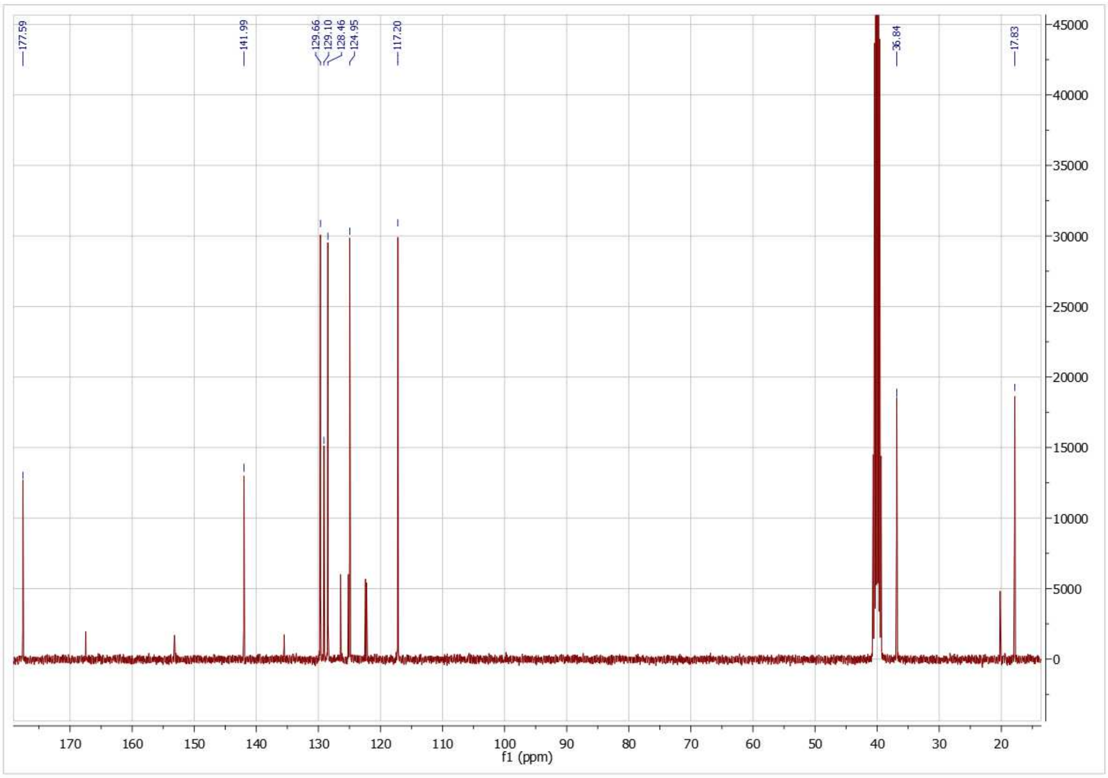

Synthesis of sodium (*E*)-3,3'-((4-(2-(3-methylbenzo[*d*]thiazol-3-ium-2-yl)vinyl)phenyl)azanediyl) dipropionate iodide

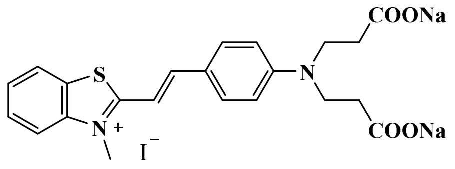

Chemical Formula: $C_{22}H_{21}IN_2Na_2O_4S$
Molecular Weight: 582,3640

Piperidine (33.93 mL, 0.34 mmol, M = 85.15 g/mol, d = 0.86 g/mL) was added into a mixture of 2,3-dimethylbenzo[*d*]thiazol-3-ium iodide (1.00 g, 3.43 mmol, M = 291.15 g/mol) in methanol (20 mL) and 3,3'-((4-formylphenyl)azanediyl)dipropionate (1.06 g, 3.43 mmol, M = 309.23 g/mol) at room temperature, and then the solution was refluxed overnight. After cooling to room temperature, a pure red-brown compound was obtained (1.56 g, 78.0% yield) by filtering and washing with cold methanol and then with diethyl ether.

^{1}H NMR (400 MHz, D_2O) δ 7.60 (s, 1H), 7.37 (s, 2H), 7.17 (m, 2H), 6.85 (s, 2H), 6.50 (m, 1H), 5.81 (s, 2H), 3.46 (s, 3H), 3.07 (s, 4H), 2.22 (s, 4H).

^{13}C NMR (101 MHz, D_2O) δ 179.74 (s), 169.62 (s), 151.10 (s), 148.96 (s), 140.61 (s), 128.90 (s), 127.51 (s), 126.26 (s), 122.92 (s), 119.94 (s), 114.49 (s), 111.02 (s), 103.41 (s), 47.20 (s), 44.57 (s), 35.12 (s), 34.32 (s), 22.23 (s).

HRMS (ESI MS) m/z: theor: 582.3640 found: 583.1372 ($[M+H]^+$ detected)

^{1}H NMR spectrum of sodium (*E*)-3,3'-((4-(2-(3-methylbenzo[*d*]thiazol-3-ium-2-yl)vinyl)phenyl)azanediyl)dipropionate iodide

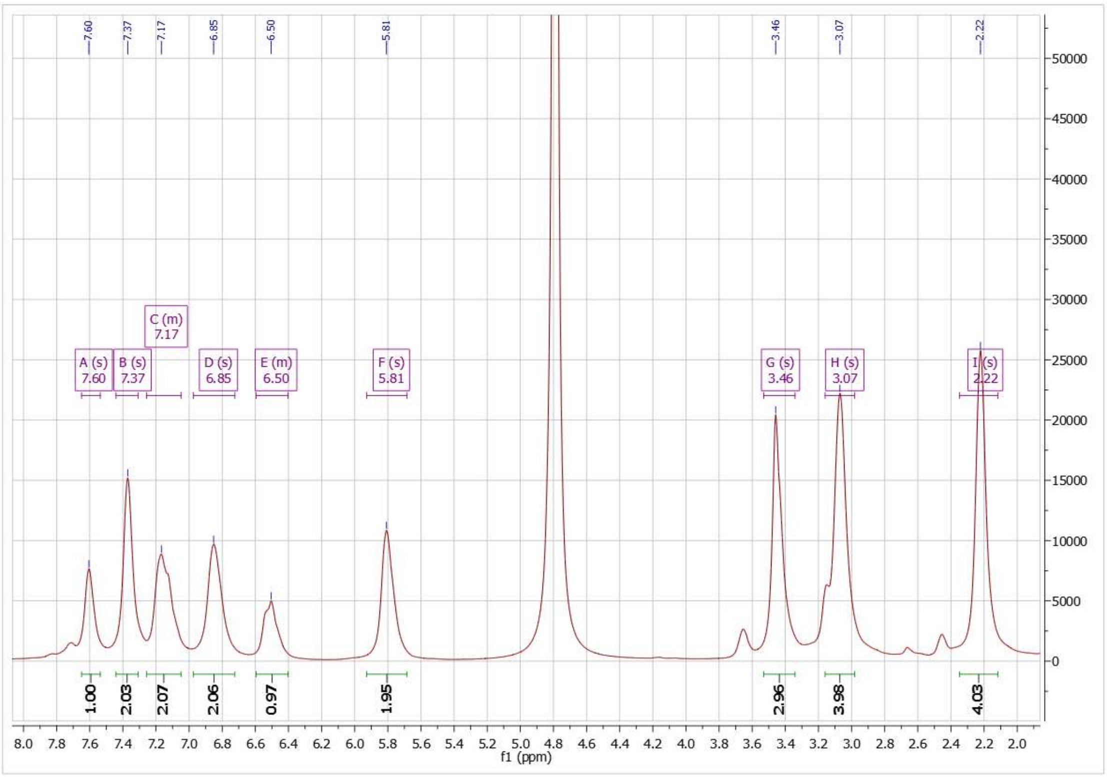

^{13}C NMR spectrum of sodium (*E*)-3,3'-((4-(2-(3-methylbenzo[*d*]thiazol-3-ium-2-yl)vinyl)phenyl)azanediyl)dipropionate iodide

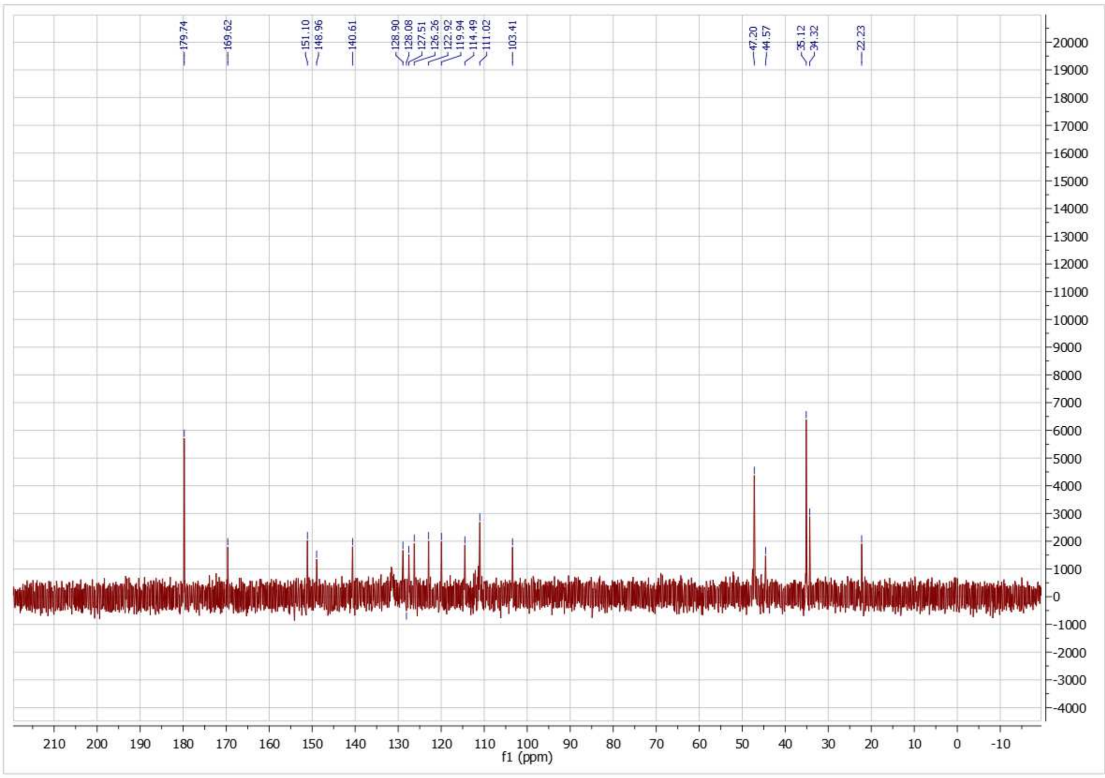

Supplement: Supplementary file 1 — Supporting Information [file SMLL-21-2411888-s001.pdf]
